# Beyond conventional lithium-ion: anode-free lithium metal batteries for ultra-high energy and greener storage solutions

**DOI:** 10.1039/d6ra01751g

**Published:** 2026-05-26

**Authors:** Zewdu Tadesse Wondimkun, Haojie Fei, Nikhitha Joseph, Petr Sáha

**Affiliations:** a Centre of Polymer Systems, Tomas Bata University in Zlín 76001 Zlín Czech Republic wondimkun@utb.cz; b University Institute, Tomas Bata University in Zlín 76001 Zlín Czech Republic; c Department of Chemistry, College of Natural and Computational Science, Debre Brehan University Debre Berhan Ethiopia

## Abstract

Lithium metal batteries (LMBs) are widely recognized as promising next-generation energy storage systems for electric vehicles and portable electronics owing to their exceptionally high theoretical energy density and specific capacity. However, their practical implementation is fundamentally constrained by severe interfacial and morphological instabilities, including lithium dendrite growth, dead lithium formation, continuous parasitic side reactions, and low Coulombic efficiency during cycling. These coupled degradation processes lead to rapid capacity fading and significant safety concerns. Anode-free lithium metal batteries (AFLMBs), which rely on *in situ* lithium plating onto a bare current collector and eliminate excess lithium metal, have emerged as an attractive configuration to maximize cell-level energy density while reducing material cost and simplifying fabrication. Nevertheless, the absence of a lithium reservoir renders AFLMBs highly sensitive to irreversible lithium loss. Limited cycle life and rapid capacity decay primarily originate from unstable solid electrolyte interphase (SEI) formation, current collector surface heterogeneity, and uncontrolled lithium nucleation and growth during repeated plating/stripping processes. This review systematically summarizes the fundamental working principles of AFLMBs and critically examines the key scientific and technical challenges limiting their practical application. Particular emphasis is placed on mechanistic insights into lithium deposition and stripping behavior, dendrite initiation and propagation, and dead lithium accumulation from the perspectives of electrochemical kinetics, ion transport, interfacial chemistry, and mechanical stability. Strategies to enhance AFLMB performance including electrolyte engineering, surface modification and structural design of current collectors, and optimization of cycling protocols—are comprehensively discussed. Their effects on interfacial stability, Coulombic efficiency, lithium utilization, and overall electrochemical performance are critically evaluated. Finally, future perspectives and research directions are proposed, highlighting the necessity of advanced characterization techniques, quantitative evaluation metrics, and validation under practical operating conditions. Through rational material design and interfacial engineering, AFLMBs hold substantial promise for enabling safer, high-energy-density, and commercially viable next-generation energy storage technologies.

## Abstract 2

Anode-free lithium metal batteries achieve stable cycling through controlled Li deposition and nucleation. Electrolyte design, cycling protocols, and surface modifications enable uniform Li^+^ flux and robust artificial SEI formation.

## Graphical abstract

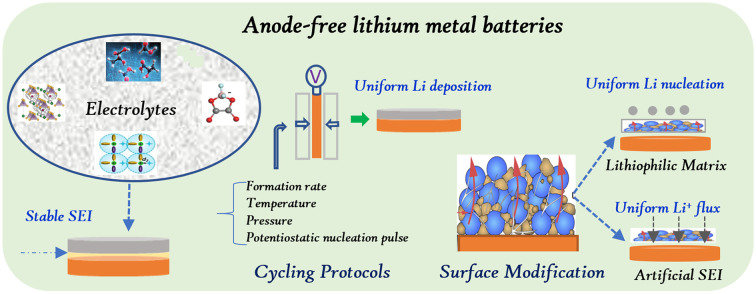

## 1 Introduction

Global energy demand is rapidly increasing due to modernization and technological advancement, creating significant challenges for sustainable energy supply. Therefore, the development of alternative energy sources and efficient energy storage systems is essential. In particular, high-energy-density, stable, cost-effective, and environmentally friendly storage technologies are critical for next-generation applications such as portable electronics and electric vehicles.^1–4^

Lithium-ion batteries (LIBs) achieved widespread commercial success in the 1990s; however, their intercalation-based chemistry limits their energy density to ∼300 Wh kg^−1^. In contrast, lithium metal batteries (LMBs) provide a pathway to higher energy density due to the ultrahigh theoretical capacity (3860 mAh g^−1^) and low electrochemical potential (−3.04 V *vs.* SHE) of lithium metal.^5–9^

Despite these advantages, LMBs face critical challenges, including parasitic reactions with electrolytes, unstable solid electrolyte interphases (SEI), and lithium dendrite growth, which lead to low Coulombic efficiency and safety risks such as internal short circuits. Additionally, the use of thick lithium foils increases cost and reduces practical energy density.^10–12^

Various strategies such as 3D current collectors, interfacial engineering, protective coatings, solid-state electrolytes, and electrolyte additives have been explored to mitigate these issues. However, many approaches focus primarily on cycling stability while overlooking energy density and lightweight design, limiting their practical impact.^5,13,14^

**Fig. 1:**
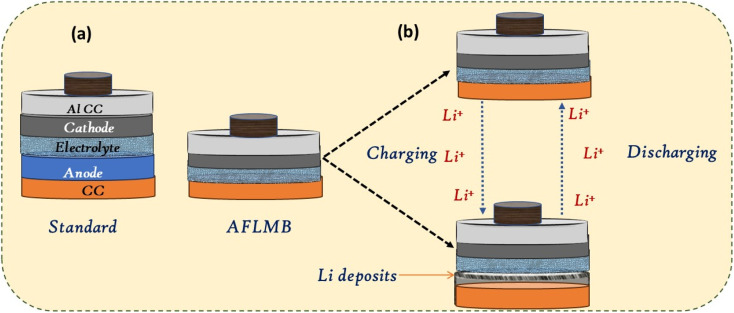
Schematic illustrations of (a) standard lithium battery and AFLMB configurations, and (b) working principles of AFLMBs. Adapted with permission from ref. 15 (ACS, © 2021).

To overcome these limitations, anode-free lithium metal batteries (AFLMBs) have emerged as a promising alternative. In AFLMBs, the anode consists of a bare current collector, with lithium supplied from the cathode (*e.g.*, LFP, NMC, NCM) (Fig. 1a).^15^ This design enables higher energy density, simplified fabrication, and reduced material cost compared to conventional LMBs.^2,16–19^

This review provides an overview of AFLMBs, focusing on their working principles, advantages, and key challenges, including energy density, cycling stability, and safety. Recent advances and characterization techniques are discussed, followed by perspectives on future research directions to accelerate the development of practical AFLMB systems.

## 2 Basic principles of anode-free batteries

Substituting conventional graphite anodes is a key strategy for improving battery energy density. In traditional LIBs, the anode significantly limits performance (∼200 Wh kg^−1^, 600 Wh L^−1^). Although approaches such as thin-film lithium batteries have been explored, their high cost and safety concerns restrict practical application.^20–23^

Using lithium metal anodes can increase energy density but introduces critical issues such as dendrite growth, low cycle life, and safety risks. To address these limitations AFLMBs have emerged as a promising alternative.^24–26^

AFLMBs eliminate lithium metal at the anode during assembly, Lithium is instead supplied from the cathode, resulting in an anode-to-cathode (N/P) ratio approaching zero. This design reduces anode mass and volume, simplifies cell architecture, and improves energy density.^7,27,28^ In a typical AFLMB, during charging, lithium ions are deposited *in situ* onto the copper surface as a thin lithium layer (Fig. 1b).

### 2.1 Key advantages and challenges of anode-free lithium metal batteries

#### 2.1.1 Merits of anode-free lithium metal batteries

Recently developed AFLMBs eliminate lithium metal at the anode during assembly, improving safety by avoiding lithium handling and increasing energy density *via* better utilization of cathode lithium. Consequently, AFLMBs are a promising alternative for next-generation energy storage systems.^29–33^ Their key advantages are as follows:

(a) High energy density: by eliminating excess lithium and host anode materials, AFLMBs significantly reduce anode mass and volume, enabling ∼25–40% higher gravimetric and ∼15–30% higher volumetric energy density compared to conventional systems.^34–39^

(b) Improved safety: AFLMBs are assembled without free lithium, reducing parasitic reactions, thermal risks, and the likelihood of short circuits, thereby enhancing operational safety.^40,41^

(c) Reduced cost: removing lithium metal and simplifying processing lowers material and manufacturing costs (up to ∼15%), while enabling compatibility with existing LIB production lines.^42,43^

(d) Capacity loss mechanism analysis: the absence of excess lithium allows more accurate analysis of lithium loss and degradation mechanisms, improving understanding of battery aging.^44,45^

(e) Simplified manufacturing: AFLMBs eliminate anode fabrication steps, reducing process complexity, cost, and production time, while improving scalability.^37,41^

Table 1 summarizes the key advantages of AFLMBs over conventional LMBs. The elimination of the Li metal anode (30–50 µm, ≈4–10 mg cm^−2^) significantly reduces anode thickness and mass, resulting in a ∼65–75% decrease in anode mass (from ∼10–12 to ∼3–4 mg cm^−2^) while retaining the same Cu current collector.^46–48^ Consequently, the inactive mass fraction decreases from ∼25–35% to ∼15–20%, improving overall cell efficiency.^49^ This reduction enables higher gravimetric (400–480 Wh kg^−1^) and volumetric (900–1050 Wh L^−1^) energy densities, corresponding to increases of ∼25–40% and ∼15–30%, respectively.^50^ However, the capacity remains cathode-limited (∼3.0 mAh cm^−2^), indicating that these gains arise from improved mass utilization rather than increased capacity.

**Table 1:** Key quantitative advantages of AFLMBs over LMBs

Parameter	LMB	AFLMB	Quantitative advantage	References
Initial anode thickness	30–50 µm Li foil	0 µm (no Li foil)	↓ eliminates 30–50 µm Li (∼4–10 mg cm^−2^)	46
Anode current collector	Cu (≈8 µm) + Li	Bare Cu (≈8 µm)	Same Cu, but no Li	47
Anode mass	∼10–12 mg cm^−2^ (Cu + Li)	∼3–4 mg cm^−2^ (Cu only)	↓ ∼65–75% reduction	48
Inactive mass fraction	25–35% of total cell	15–20% of total cell	↓ reduces by ∼30–40%	49
Gravimetric energy density (cell level)	300–350 Wh kg^−1^	400–480 Wh kg^−1^	↑ +25–40%	50
Volumetric energy density (cell level)	700–800 Wh L^−1^	900–1050 Wh L^−1^	↑ +15–30%	50
Specific capacity (cell-level)	∼3.0mAh cm^−2^ (depends on cathode)	∼3.0 mAh cm^−2^ (same cathode)	Same cathode-limited	47
Cost saving (material only)	Li foil: $60–100 kg^−1^ → ≈$0.1–0.2 per cell	Cu foil only	↓ $0.10–0.20 per cell	47
Manufacturing complexity	Requires dry-room Li handling	Compatible with standard LIB lines	↓ ∼20–30% lower production cost	47
Safety	Higher short-circuit risk due to thick Li	Lower Li inventory → lower risk	Safer at cell rupture/failure	47

From a practical standpoint, AFLMBs simplify manufacturing by eliminating lithium metal handling, enabling compatibility with existing lithium-ion battery production lines and reducing costs by ∼20–30%, along with material savings of ∼$0.10–0.20 per cell.^47^ The reduced lithium inventory also improves safety by lowering the risk of severe short circuits under failure conditions. However, these advantages depend critically on efficient lithium utilization and high Coulombic efficiency. In the absence of excess lithium, any irreversible lithium loss directly reduces active lithium, making AFLMBs highly sensitive to interfacial instability. Therefore, despite the clear system-level benefits outlined in Table 1, their practical viability relies on addressing challenges such as non-uniform Li deposition, dead lithium formation, and unstable SEI behavior.

#### 2.1.2 Challenges of anode-free lithium metal batteries

Despite their high energy density and simplified configuration, AFLMBs face significant challenges that limit their practical application. These challenges stem primarily from the absence of a lithium reservoir and the reliance on repeated Li plating/stripping on a bare Cu current collector (CC), which exhibits lithiophobicity and surface heterogeneity.^38,51–54^ As summarized in Table 2, these issues manifest through several interrelated failure mechanisms.

**Table 2:** Mechanism-driven summary of key failure pathways in anode-free lithium metal batteries

Failure mechanism	Key issue	Quantitative indicators	Severity	References
Dendrite growth	Non-uniform Li deposition leading to internal short circuits	CE: ∼98–99%; cycle life: <100 cycles (baseline Cu)	High	55
SEI instability	Continuous fracture/reformation of SEI causing electrolyte depletion	CE decay: >2% per cycle; high interfacial resistance	High	56
Dead lithium formation	Electrically isolated (“dead”) Li causing irreversible capacity loss	Irreversible capacity: 2–5% per cycle	Medium	57
High nucleation overpotential	Poor lithiophilicity of Cu leading to delayed and uneven Li nucleation	Overpotential: 50–100 mV (bare Cu)	Moderate	58
Uneven Li-ion flux/hotspots	Localized current density inducing heterogeneous Li deposition	Capacity retention: <80% after 50 cycles	High	59
First-cycle Li loss (AFLMB-specific)	Irreversible Li consumption due to SEI formation without Li reservoir	Initial CE: ∼85–95%	High	60

Table 2 summarizes the dominant failure mechanisms in AFLMBs in order of their impact on cell degradation. Dendrite growth is identified as a critical failure mode, driven by non-uniform lithium deposition, leading to rapid short circuit risks and limited cycle life (<100 cycles on bare Cu) with only moderate Coulombic efficiency (∼98–99%).^55^ Closely coupled with this, SEI instability results in continuous interfacial degradation, electrolyte consumption, and resistance buildup, as reflected by CE decay exceeding 2% per cycle.^56^

Dead lithium formation represents a secondary but cumulative loss pathway, contributing to irreversible capacity loss (2–5% per cycle) due to electrically isolated lithium.^57^ At the nucleation stage, high overpotential on bare Cu (50–100 mV) indicates poor lithiophilicity, which promotes uneven lithium deposition and triggers downstream degradation processes.^58^

At the mesoscale, uneven Li-ion flux and localized hotspots further exacerbate deposition heterogeneity, leading to rapid capacity fading (<80% retention within 50 cycles).^59^ Finally, a key AFLMB-specific limitation is first-cycle lithium loss, where irreversible SEI formation results in low initial Coulombic efficiency (∼85–95%), directly depleting the limited lithium inventory.^60^ Overall, the table highlights that interfacial instability and deposition heterogeneity are the primary drivers of failure, with nucleation behavior and lithium inventory loss acting as critical initiating factors.

To address these challenges, current research focuses on strategies such as lithiophilic surface engineering of CCs, electrolyte optimization, and cycling protocol regulation to achieve uniform Li deposition, stabilized SEI, and improved reversibility.

## 3 Strategies to enhance safety, cycling performance, and energy density

**Fig. 2:**
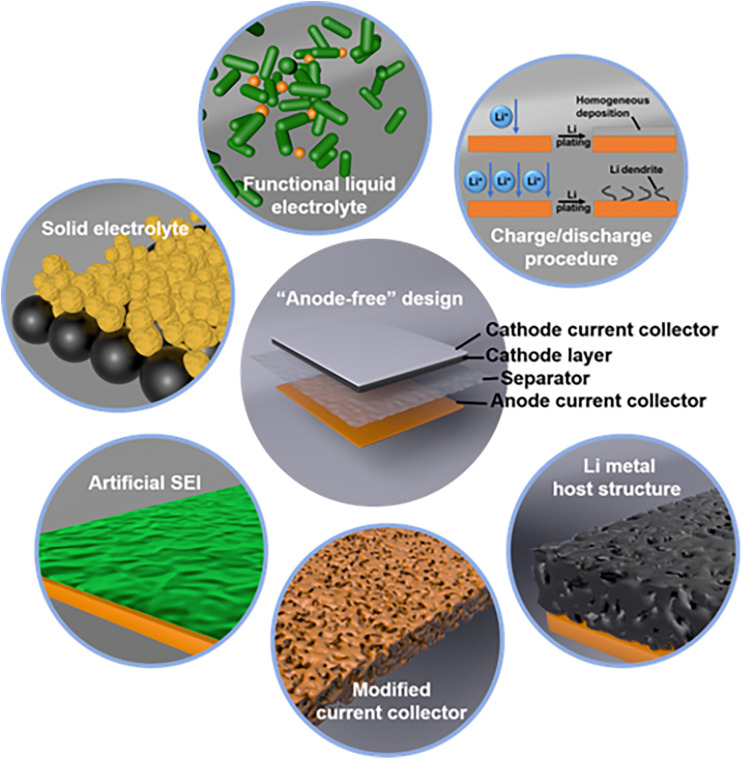
Strategies for AFLMBs. Approaches include functional or solid electrolytes, current collector (CC) surface modifications, artificial SEI layers to suppress nonuniform Li deposition, Li host structures on the CC, and tailored charge–discharge protocols to control Li plating rates, reproduced with permission from ref. 50 (Wiley, 2021, CC BY 4.0).

The bare Cu surface is inherently rough, lithiophobic, and heterogeneous, resulting in a high nucleation barrier and large overpotential for Li plating, which promotes dendritic growth. To address this, controlling Li deposition to achieve uniform and compact layers is essential. Recent research has focused on three main strategies: surface/interface modification, electrolyte optimization, and cycling parameter design (Fig. 2), which are summarized below.

### 3.1 Surface modification of current collectors

The electrolyte–electrode interface plays a critical role in Li deposition behavior. Strategies such as introducing lithiophilic nucleation sites, engineering current collector structures, and applying artificial SEI layers can reduce nucleation overpotential and regulate Li^+^ flux, leading to more uniform Li growth and improved cycling stability.^61–64^ In addition, advanced electrode architectures, such as soluble-salt-template-derived two-/three-dimensional nanosheet frameworks, can homogenize current distribution and minimize local current density fluctuations. These porous, high-surface-area structures provide abundant nucleation sites, promote uniform lithium plating, and accommodate volume changes during cycling. This highlights that effective surface modification must integrate both chemical lithiophilicity and structural design to control lithium deposition behavior across multiple length scales.^65^ Therefore, the synergistic integration of interfacial engineering and structural design is essential to achieve stable and uniform lithium deposition in AFLMBs.

### 3.2 Electrolyte formulations

Electrolytes govern ion transport and electrochemical stability. Tailoring electrolyte composition (*e.g.*, high-concentration systems and functional additives) can homogenize Li^+^ flux, stabilize interfacial reactions, and improve plating/stripping efficiency, resulting in better cycle life and safety.^3,66–70^

### 3.3 Cycling parameter design

Optimizing operating conditions such as formation protocols, voltage windows, current density, temperature, and stack pressure helps stabilize SEI formation, reduce side reactions, and enhance cycling stability.^71–76^

**Fig. 3:**
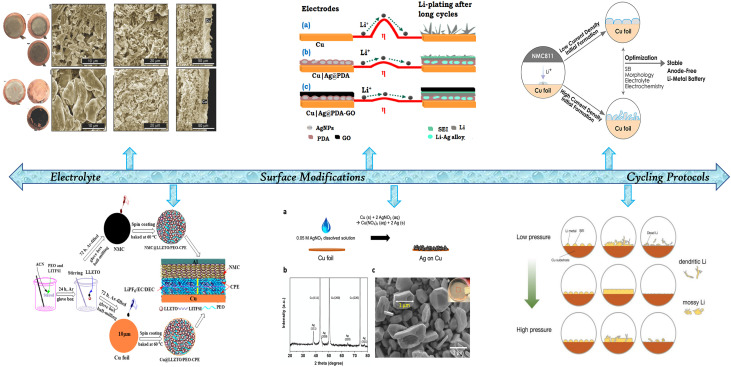
Schematic summary of selected studies on interface modification, reproduced with permission from ref. 77 (Wiley, © 2016) and ref. 78 (ACS, © 2020); electrolyte optimization, reproduced with permission from ref. 13 (Elsevier, © 2021) and ref. 79 (ACS, © 2022); and cycling parameter design, reproduced from ref. 80 (ACS, 2016, CC BY 4.0) and reproduced with permission from ref. 81 (Wiley, © 2024).

These strategies collectively mitigate dendrite growth, stabilize the SEI, and improve overall battery performance. Representative studies are summarized in (Fig. 3).

#### 3.1.1 Surface modification of the current collectors in AFLMBs

The current collector (CC) serves as an electron pathway and structural support. In AFLMBs, surface modification of CCs is essential to regulate Li nucleation and suppress dendrite growth. Approaches such as lithiophilic coatings and artificial SEI layers can improve interfacial stability, enhance ionic conductivity, and increase mechanical robustness, leading to better cycling performance.^82–88^

##### 3.1.1.1 Lithiophilic material coatings on current collectors in AFLMBs

Surface modification using lithiophilic materials is an effective strategy to lower nucleation overpotential and promote uniform Li deposition. These materials act as nucleation seeds with strong Li affinity and high electronic conductivity, thereby suppressing dendrite formation and improving cyclability. Common lithiophilic materials include metals (*e.g.*, Sn, Zn, Ag, Au) and metal oxides or nanostructured materials, which provide favorable Li nucleation sites. In addition, nanostructured coatings such as ZnO quantum dots, Ag nanoseeds, and doped carbon materials further enhance lithiophilicity and interfacial stability.^18,30,31,54,79,89–94^ The following sections focus on representative coatings such as Sn-, Zn-, Ag-, and liquid metal-based systems.

###### 3.1.1.1.1 Sn-based lithiophilic materials

**Fig. 4:**
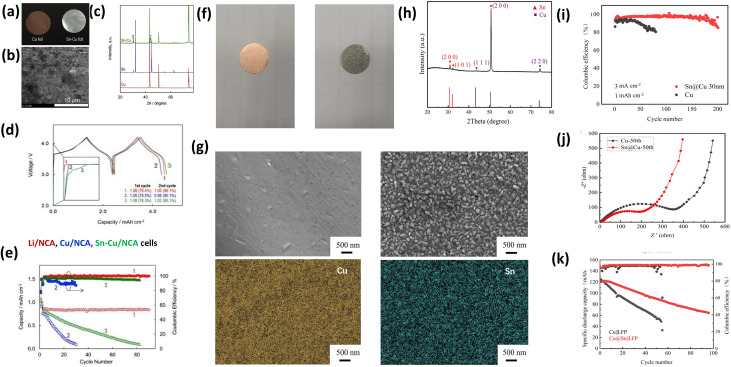
Sn-based lithiophilic coatings on Cu CCs. (a) Digital images of Cu and Sn–Cu foils; (b) SEM image of the Sn–Cu foil surface; (c) XRD patterns of Cu, Sn, and Sn–Cu foils; (d) the 1st and 2nd voltage profiles with discharge capacity (mAh cm^−2^) and CE); (e) discharge capacity and CE *versus* cycle number, reproduced with permission from ref. 96 (Elsevier, © 2017); (f) digital images of Cu and Cu@Sn foils; (g) XRD patterns of Cu@Sn foil; (h) SEM images and corresponding EDS mapping of Cu and Cu@Sn foils; (i) cycling performance of Li‖Cu and Li‖Cu@Sn cells at 3 mA cm^−2^ and 3 mAh cm^−2^; (j) EIS of Li‖Cu and Li‖Cu@Sn cells after 50 cycles; (k) cycling performance of Cu‖LFP and Cu@Sn‖LFP cells at 0.1C, reproduced with permission from ref. 97, (Elsevier, © 2024).

Tin(Sn) coatings have been widely investigated for Cu current collectors in AFLMBs due to their ability to form Li–Sn alloys, which enhance electrical conductivity, lithiophilicity, and interfacial adhesion, thereby reducing nucleation overpotential and promoting uniform Li deposition.^95^ Compared to many other lithiophilic coatings (*e.g.*, Zn-, Ag-, or carbon-based systems), Sn is particularly effective due to its ability to alloy with Li, providing both chemical affinity and dynamic Li accommodation during cycling, which is critical for mitigating localized Li nucleation. S. S. Zhang *et al.*^96^ demonstrated an electroless Sn coating on Cu foil, forming a dense and homogeneous layer confirmed by SEM (Fig. 4a and b) and XRD (Fig. 4c). Electrochemical evaluation in Li/NCA cells showed improved capacity retention and Coulombic efficiency (CE), with performance ranked as Li/NCA > Sn–Cu/NCA > Cu/NCA (Fig. 4d and e). Notably, the reduction in nucleation overpotential was directly correlated with more uniform Li plating, as evidenced by the morphological observations in Fig. 4a and b, suggesting that Sn modifies the initial nucleation thermodynamics rather than only improving conductivity. The Li–Sn alloy acted as an “electrical glue,” enhancing Li adhesion and suppressing dendritic growth, which is a key mechanism underpinning improved cycling stability.

Y. Li *et al.*^97^ further showed that a crystalline Sn nanolayer (Cu@Sn) significantly enhances electrochemical performance. The uniform nanoparticle coverage was confirmed by SEM (Fig. 4f), XRD (Fig. 4g), and EDS mapping (Fig. 4h). Li‖Cu@Sn cells exhibited high CE (>95%) over 200 cycles at 1 mA cm^−2^ and stable cycling at higher current density (3 mA cm^−2^), outperforming bare Cu (Fig. 4i). Importantly, electrochemical impedance analysis demonstrated improved Li^+^ transport kinetics on the Sn-coated surface (Fig. 4j), indicating that Sn coatings influence both interfacial charge transfer and ion diffusion. AFLMBs using Cu@Sn‖LFP also achieved an initial areal capacity of 121 mAh g^−1^ with 95.4% CE and sustained performance over 45 cycles, whereas Cu‖LFP cells degraded rapidly (Fig. 4k).

Sn-based lithiophilic coatings enable uniform Li nucleation, dense dendrite-free deposition, and better interfacial adhesion, reducing overpotentials and enhancing cycling stability through Li–Sn alloy formation and improved kinetics. However, they have limited buffering capacity for high capacities and long-term cycling, and while simple and scalable compared to 3D hosts, further optimization is needed for practical AFLMB use.

###### 3.1.1.1.2 Ag-based lithiophilic materials

Recent studies have explored lithium-wetting metals such as Ag as coatings on CCs to enhance Li deposition. Li readily alloys with Ag, forming nuclei with high binding energy that lower nucleation overpotential and promote uniform Li growth. Owing to its high conductivity and low alloying potential (0–0.25 V *vs.* Li^+^/Li), Ag is considered a promising candidate for AFLMBs.^98,99^ However, its effectiveness strongly depends on structural design (*e.g.*, dispersion, porosity) and operating conditions, which are often overlooked.

**Fig. 5:**
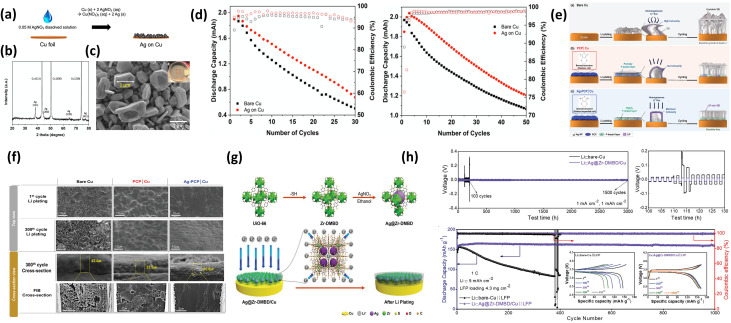
Ag-based lithiophilic surface engineering on Cu CCs. (a) Schematic illustration of Ag particle formation *via* the reaction between Cu foil and 0.05 M AgNO_3_ solution; (b) XRD pattern of Ag deposited on Cu; (c) top-view SEM image of Ag particles (magnified region highlighted in the inset), showing particle size distribution; (d) cycling performance and GCD curves of anode-free LiFePO_4_ cells using pristine Cu and Ag/Cu in 1.0 M LiPF_6_ (EC/EMC, v/v 3 : 7) and 1.0 M LiTFSI (DOL/DME, v/v 5 : 5) electrolytes, reproduced with permission from ref. 79 (ACS, © 2022); (e) schematic illustration of Li plating behavior and SEI formation mechanisms on bare Cu, PCP-Cu, and Ag-PCP-Cu; (f) SEM (top-view and cross-sectional) and FIB images showing Li deposition behavior on bare Cu, PCP-Cu, and Ag-PCP-Cu after plating 3 mAh cm^−2^ at 3 mA cm^−2^ (first and 300th cycles), reproduced with permission from ref. 100 (Wiley, © 2022); (g) schematic of Ag-impregnated Zr-DMBD MOF fabrication *via* Ag–S coordination chemistry and Li^+^ transport through polar mercapto ligands into MOF pores containing lithiophilic Ag nanoparticles; (h) electrochemical performance of Li⊂Ag@Zr-DMBD/Cu symmetric cells and Li⊂Ag@Zr-DMBD/Cu‖LFP full cells, reproduced with permission from ref. 101 (Wiley, © 2023).

W. Shin *et al.* prepared Ag particles on Cu *via* an ion–exchange reaction (Fig. 5a–c).^79^ The Ag/Cu electrode improved CE (89.3% → 95.2% in carbonate; 93.5% → 99.5% in ether) and reduced nucleation overpotential (158 → 63 mV) (Fig. 5d). These results confirm that Ag primarily mitigates the high nucleation barrier, but does not fully resolve SEI instability or dead Li formation, especially in carbonate electrolytes.

S. Pyo *et al.* combined Ag nanoparticles with a p-doped conjugated polymer (Ag-PCP), forming a porous 3D structure that reduces local current density and stabilizes the SEI (Fig. 5e and f).^100^ The electrode delivered low polarization (∼17 mV) and stable cycling over 300 cycles. Compared to simple Ag coatings, this design simultaneously addresses nucleation heterogeneity, SEI instability, and volume change, highlighting the importance of multifunctional architectures.

X. Li *et al.* introduced a porous MOF-based host (Ag@Zr-DMBD (Fig. 5g and h); to enable compartmentalized Li storage.^101^ The electrode sustained uniform Li deposition up to 5 mAh cm^−2^ with minimal thickness change and long cycling stability. This demonstrates that porous Ag-based hosts are more effective in mitigating dead Li accumulation and volume expansion under practical areal capacities.

A cross-comparison indicates that planar Ag coatings mainly reduce nucleation barriers, while composite and porous structures provide additional control over SEI stability and Li accommodation. Thus, performance improvement correlates with increasing structural complexity and multifunctionality. Ag-based modifications improve Li nucleation and interfacial stability. However, their practical effectiveness relies on synergistic integration with structural and interfacial design, rather than acting as a standalone solution.

###### 3.1.1.1.3 Zn-based lithiophilic materials

Zinc(Zn) is a cost-effective lithiophilic metal capable of forming Li–Zn alloys at room temperature, enabling its use as a nucleation-regulating interlayer on Cu CCs. This reduces the nucleation energy barrier and promotes more uniform Li deposition.^102^ However, the effectiveness of Zn is highly dependent on coating thickness, phase composition, and interfacial stability during cycling.

**Fig. 6:**
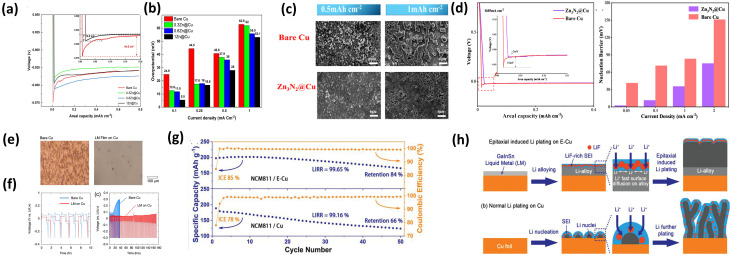
(a) Lithium plating/stripping curves of bare Cu, 0.3Zn@Cu, 0.6Zn@Cu, and 1Zn@Cu cells; (b) Li nucleation overpotentials of the same electrodes at various current densities, reproduced from ref. 2 (2024, CC BY 4.0); (c) SEM images of bare Cu after Li plating at different capacities; (d) voltage–capacity curves and Li nucleation barriers on Cu and Zn_3_N_2_@Cu at 0.05 mA cm^−2^, reproduced with permission from ref. 29 (American Chemical Society, © 2023); (e) optical microscopy of Cu before (left) and after (right) LM coating; (f) half-cell Li plating/stripping profiles comparing LM-coated Cu and bare Cu, reproduced from ref. 63 (2022, CC BY 4.0); (g) cycling performance of NCM811/E-Cu (top) and NCM811/Cu (bottom) pouch cells; (h) schematic of epitaxial-induced Li plating on E-Cu *versus* Cu, reproduced with permission from ref. 106 (Wiley, © 2021).

P. Afzali *et al.*^2^ electrodeposited Zn layers with controlled thicknesses (300 nm–1 µm) onto Cu (Fig. 6a). All Zn@Cu electrodes showed significantly reduced nucleation overpotentials (∼17–19 mV) compared to bare Cu, with further reduction observed at higher Zn loading (Fig. 6b). These results confirm that Zn primarily mitigates the nucleation barrier through Li–Zn alloy formation, which increases active sites and reduces local current density. However, the marginal difference between thicknesses suggests diminishing returns beyond a certain loading. The Li–Zn alloy (Li_1_Zn_1_) improves interfacial contact and Li-ion transport, resulting in smoother Li morphology and improved cycling stability. Nevertheless, excessive Zn may introduce additional mass and potential mechanical instability during repeated alloying/dealloying.

Similarly, Y. Zhu *et al.*^29^ introduced a Zn_3_N_2_ interlayer on Cu to further regulate Li deposition. Zn_3_N_2_@Cu exhibited lower polarization (37.2 mV *vs.* 102.7 mV for Cu) and stable CE (>99.3% over 230 cycles), while bare Cu rapidly degraded (Fig. 6c and d). Compared to metallic Zn, Zn_3_N_2_ provides enhanced interfacial stability and reduced resistance, indicating that compound-based interlayers can better address SEI instability in addition to nucleation issues. In full cells, Zn_3_N_2_@Cu‖LFP significantly outperformed Cu‖LFP, retaining 45.6% capacity after 100 cycles *versus* 3.6% for bare Cu. Post-cycling analysis confirmed reduced dead Li and smoother morphology.

Metallic Zn lowers Li nucleation barriers, while Zn-based compounds (*e.g.*, Zn_3_N_2_) enhance interfacial stability and suppress dead Li. Performance depends on both composition and structure. Key limitations include parasitic reactions, structural degradation, and limited validation under practical conditions (high areal capacity, lean electrolyte). Effective Zn-based interlayers require a balance of alloying behavior, interfacial stability, and structural robustness not just higher Zn loading.

###### 3.1.1.1.4 Liquid metal (LM)coating-based lithiophilic materials

Liquid metals serve as adaptive lithiophilic interlayers due to their high conductivity, fluidity, and self-healing properties, which accommodate volume changes and suppress dendrite formation. Upon interaction with Li, Ga-based liquid metal alloys undergo alloying to form a solid-like interphase, thereby reducing the nucleation barrier and enabling more uniform deposition. However, their performance is strongly influenced by wettability, layer stability, and compatibility with electrolytes.^103–105^

S. Koul *et al.*^63^ demonstrated that Ga-based LM coatings (Ga–In–Sn) significantly reduce overpotential and improve cycling stability. LM-coated Cu showed a ∼50% reduction in overpotential and extended cycling life (from <40 h to 180 h) (Fig. 6f). This improvement arises from the ability of LM to eliminate surface heterogeneity and redistribute current density, directly addressing uneven nucleation. In full cells with high-loading cathodes (∼6 mAh cm^−2^), LM coatings improved performance, particularly with FEC-based electrolytes. This indicates that LM effectiveness is closely coupled with electrolyte chemistry and SEI formation.

Similarly, L. Lin *et al.*^106^ applied LM onto epitaxial Cu (E-Cu), achieving dense and uniform Li deposition (37.5 µm *vs.* 46.7 µm on bare Cu) and a high initial CE (98.24%). In practical pouch cells (high loading, lean electrolyte), E-Cu improved capacity retention from 66% to 84% over 50 cycles and delivered an energy density of 420 Wh kg^−1^ (Fig. 6g and h). Notably, this study demonstrates the advantage of LM coatings under more realistic conditions, highlighting their potential for practical applications.

LM coatings reduce nucleation barriers and dynamically adapt to volume and surface changes, making them more effective than static lithiophilic coatings for long-term interfacial stability. However, challenges remain, including LM redistribution or loss during extended cycling, high material cost (*e.g.*, Ga/In), and limited scalability and stability under lean electrolyte conditions. While LM coatings offer a self-adaptive interface that enhances Li deposition and cycling stability, their practical implementation requires precise control of composition, loading, and electrolyte compatibility.

#### 3.1.2 Surface modification of the CCs with artificial SEI (buffer layer) coating material in AFLMBs

In AFLMBs, artificial SEI layers are engineered to regulate lithium plating by providing uniform chemical composition, high ionic conductivity, electrochemical stability, electronic insulation, and sufficient mechanical strength and flexibility, thereby ensuring homogeneous Li^+^ flux, suppressing side reactions, and maintaining interfacial stability. As the native SEI is typically heterogeneous, fragile, and chemically unstable, it cannot satisfy these requirements, making artificial SEI layers essential for achieving smooth, dense, and stable lithium deposition.^16,107,108^ Compared to lithiophilic coatings that primarily control nucleation, artificial SEI layers provide interfacial regulation during both Li plating and stripping.

**Fig. 7:**
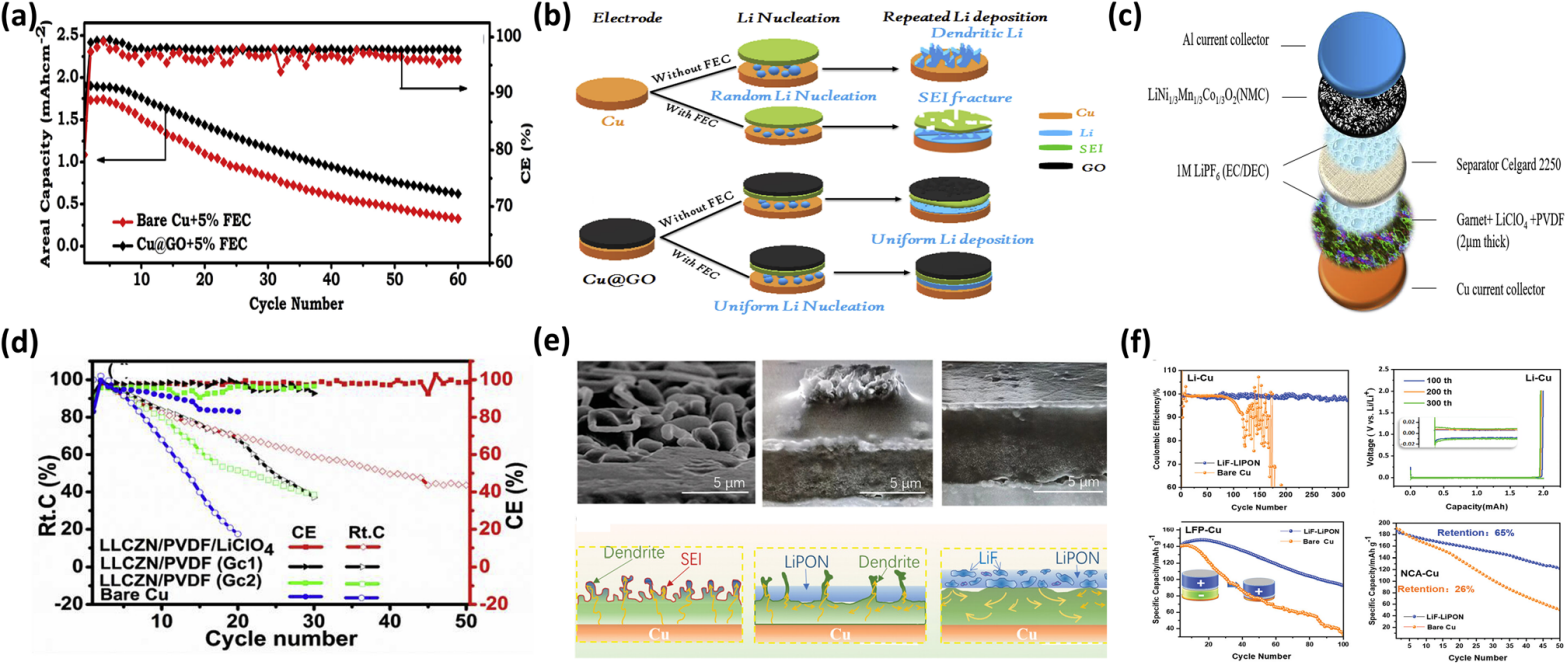
(a) Electrochemical comparison of Cu//NMC and Cu@GO//NMC at 0.5 mA cm^−2^ with 5% FEC; (b) schematic of Li nucleation and plating on bare Cu and Cu@GO with/without FEC, reproduced with permission from ref. 14 (Elsevier, © 2020); (c) configuration of an anode-free battery with garnet-composite artificial SEI; (d) electrochemical comparison of Cu@Gc1//NMC, Cu@Gc2//NMC, and Cu@Gc3//NMC at 0.2 mA cm^−2^ in 1 M LiPF_6_ EC/DEC (1 : 1 v/v) at 60 °C, reproduced with permission from ref. 109 (Wiley, © 2023); (e) cross-sectional SEM images and schematic of Li deposition on bare Cu, LiPON/Cu, and LiF–LiPON/Cu; (f) cycling performance of half-cells and anode-free full cells with bare Cu and LiF–LiPON-coated Cu under different cathodes, reproduced with permission from ref. 107 (Elsevier, © 2019).

Z.T. Wondimkun *et al.*^14^ demonstrated a binder-free, ultra-thin graphene oxide (GO) layer as an artificial SEI. On Cu, unstable native SEI leads to dendrite formation and high impedance, whereas the spin-coated GO layer stabilizes the interface. In Cu@GO//NMC full cells (with 5% FEC, 0.5 mA cm^−2^), capacity retention reached 39.87% after 50 cycles, compared to 27.94% for bare Cu, while suppressing parasitic reactions and dendritic growth (Fig. 7a and b). This improvement indicates that GO primarily acts as a physical and chemical barrier, reducing side reactions and promoting uniform Li deposition.

L.H. Abrha *et al.*^109^ developed a Li-ion-conducting composite SEI composed of garnet LLZCN, PVDF, and LiClO_4_. This structure formed an inorganic-rich SEI (LiF and LiCl), enhancing mechanical stability and interfacial integrity. In Cu@Gc3//NMC full cells, capacity retention reached 58.66% with an average CE of 97.6% after 30 cycles, while Li‖Cu@Gc3 half-cells showed reduced voltage hysteresis (20–21 mV *vs.* 35–55 mV for bare Cu) (Fig. 7c and d). These results highlight that garnet-based SEIs improve both ionic transport and mechanical durability, though long-term stability remains a challenge.

Sun *et al.*^107^ reported a LiF–LiPON composite SEI fabricated *via* vacuum thermal evaporation. Embedding LiF nanocrystals within LiPON enhanced mechanical strength and ionic conductivity, increasing fracture toughness and enabling uniform Li deposition. Li/Cu cells with this coating showed a >400% increase in cycle life, while high-loading LFP full cells improved capacity retention from 25% to 66% after 100 cycles (Fig. 7e and f). The enhanced performance arises from the synergistic effect of ionic conductivity (LiPON) and mechanical reinforcement (LiF), which suppress dendrite penetration and stabilize the interface.

Artificial SEI coatings including graphene oxide, garnet-based composites, and LiF–LiPON heterostructures effectively compensate for the limitations of native SEI by stabilizing the interface, suppressing dendrites, and enhancing ionic transport and mechanical strength. Compared to lithiophilic coatings, these buffer layers primarily regulate interfacial stability rather than nucleation, offering a complementary strategy for improving Coulombic efficiency and cycle life. However, challenges related to scalability, interfacial resistance, and long-term durability under high areal capacities remain, requiring further optimization.

#### 3.1.3 Multiple protective layers: combined lithiophilic and artificial SEI coatings in AFLMBs

To date, limited studies have systematically compared multilayer coatings with single-layer modifications to evaluate the synergistic effects of combining lithiophilic materials and artificial SEI (buffer) layers on current collectors in AFLMBs. Such multilayer strategies are expected to address two key failure mechanisms: non-uniform Li nucleation and unstable SEI formation. This gap underscores the need for comprehensive investigations into how the integration of these functional layers influences electrochemical performance. By coupling a lithiophilic nucleation layer with a robust artificial SEI, it is possible to regulate lithium nucleation and stabilize the electrode/electrolyte interface. However, whether this added complexity consistently improves long-term performance remains unclear. Understanding the interactions between these layers is crucial for optimizing current collector design. The synergistic effects of such multilayer strategies in AFLMBs are discussed in the following section.

**Fig. 8:**
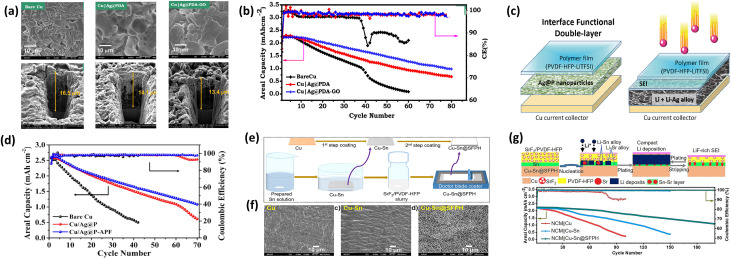
(a) SEM images of Cu electrodes after first-cycle Li deposition/stripping and corresponding FIB cross-sectional views of Li deposition; (b) cycling performance of anode-free Cu//NMC cells in 1 M LiPF_6_ EC/DEC (1 : 1 v/v) + 5% FEC at 0.5 mA cm^−2^, reproduced with permission from ref. 13 (Elsevier, © 2021); (c) schematic illustration of multilayer protective coatings on Cu current collectors; (d) cycling performance and areal capacity comparison of bare Cu//NMC, Cu/Ag@P//NMC, and Cu/Ag@P-APF//NMC at 0.2 mA cm^−2^, reproduced with permission from ref. 110 (Elsevier, © 2021); (e) fabrication process of Cu–Sn and Cu–Sn@SFPH electrodes; (f) top-view SEM images of bare Cu, Cu–Sn, and Cu–Sn@SFPH electrodes; (g) cycling stability comparison of Cu, Cu–Sn, and Cu–Sn@SFPH electrodes, reproduced with permission from ref. 111 (Elsevier, © 2024).

To enhance initial lithium nucleation in AFLMBs, Z.T. Wondimkun *et al.*^13^ introduced a dual-layer strategy by first coating lithiophilic silver nanoparticles wrapped with polydopamine (Ag@PDA) onto a lithiophobic and rough Cu substrate, followed by a graphene oxide (GO) artificial SEI layer. The Ag@PDA layer acted as nucleation seeds to promote Li–Ag alloy formation, while the GO coating regulated Li-ion diffusion and stabilized the electrode/electrolyte interface in near-zero excess Li configurations (N/P ≈ 1). As shown in Fig. 8a, the modified surface exhibits more uniform Li deposition with reduced nucleation overpotential. The two-step spin-coated electrode exhibited reduced dead Li formation, lower nucleation overpotential, and more uniform Li deposition compared with bare Cu. In anode-free Cu|Ag@PDA-GO//NMC full cells (1 M LiPF_6_ in EC/DEC (1 : 1) + 5% FEC, 0.5 mA cm^−2^), a high average Coulombic efficiency (CE) of ∼98.5% and capacity retention of ∼55.7% after 60 cycles were achieved (Fig. 8a and b). However, Fig. 8b indicates only moderate capacity retention, suggesting incomplete suppression of long-term side reactions.

Similarly, N.T. Temesgen *et al.*^110^ reported a two-step surface modification combining AgNPs coated with PDA as lithiophilic seeds and a poly(vinylidene fluoride-*co*-hexafluoropropylene) (PVDF-HFP)-based artificial protection film (APF). The Ag@PDA layer induced uniform Li–Ag alloy formation, while the APF provided mechanical robustness and regulated Li-ion flux. Compared with mossy and dendritic Li growth on bare Cu, the double-layer Cu/Ag@P/APF electrode exhibited smooth and homogeneous Li deposition. This improved morphology is evident in Fig. 8c, while Fig. 8d shows only moderate improvement in cycling stability. In anode-free Cu//NMC full cells, the modified electrode delivered a CE of 98.15% and capacity retention of 41.44% after 70 cycles at 0.2 mA cm^−2^ (Fig. 8c and d). This suggests that the polymer-based artificial SEI may introduce additional ionic resistance despite improved interfacial stability.

Expanding this multilayer concept, Hwang's group^111^ developed a dual-protective Cu–Sn@SFPH electrode incorporating lithiophilic Sn and SrF_2_ nanoparticles embedded in a PVDF-HFP matrix. The coating generated both electronically conductive (Cu–Sn/Sr alloy) and ionically conductive (LiF-rich SEI) interphases. The Sn layer formed *ex situ* Cu–Sn and *in situ* Li–Sn alloys to promote uniform nucleation, while SrF_2_ nanoparticles contributed to *in situ* formation of a LiF-rich SEI during cycling. The porous SrF_2_/PVDF-HFP framework further regulated Li-ion flux and suppressed inactive Li formation. As shown in Fig. 8e, uniform Li deposition is achieved, while Fig. 8f and g demonstrates significantly improved cycling stability and high CE. As a result, Cu–Sn@SFPH‖Li cells exhibited ultrastable cycling over 3200 h with low nucleation overpotential at 2 mAh cm^−2^. Even under lean electrolyte conditions, NCM‖Cu–Sn@SFPH pouch cells retained 72.1% capacity with an average CE of 99.9% over 120 cycles (Fig. 8e–g). Compared with Fig. 8a–d, this system shows improved durability, highlighting the role of *in situ* LiF-rich SEI formation.

Multilayer protective coatings that combine lithiophilic nucleation layers with artificial SEI buffer layers provide a rational framework for addressing multiple degradation mechanisms in AFLMBs. However, their effectiveness is highly dependent on the alignment between material selection, interfacial chemistry, and operating conditions, and they should not be assumed to universally outperform single-layer approaches. Future research should prioritize quantitative benchmarking, mechanistic studies of interlayer coupling, and validation under practical cell configurations to establish the true potential of these systems for high-energy-density applications.

### 3.2 Optimization of electrolyte formulation

Electrolytes enable ion transport and define the electrochemical stability window through the interplay of solvents, lithium salts, additives, concentration, and temperature. Key metrics energy density, cycle life, and safety depend on electrolyte formulation but require careful trade-offs. An ideal electrolyte for AFLMBs should provide high ionic conductivity, a near-unity lithium transference number, and strong electrochemical stability. However, these requirements are often conflicting, requiring balanced optimization. In anode-free systems, the absence of excess lithium necessitates ultra-high Coulombic efficiency (>99.9%), making interfacial stability critical.^17,112–116^ Thus, the electrolyte must regulate Li deposition/stripping and suppress side reactions. Electrolyte design directly addresses key failure mechanisms dendrite growth, dead lithium formation, and SEI instability. Interfacial chemistry dominates over bulk conductivity; advanced electrolytes improve stability but introduce trade-offs (*e.g.*, cost, viscosity).^117–119^ Overall, electrolyte optimization is a multi-dimensional problem involving solvation, interphase chemistry, and transport properties, and the following section discusses key strategies with emphasis on design principles and limitations.

#### 3.2.1 Electrolyte additives

The incorporation of functional additives into liquid electrolytes is an effective strategy to suppress lithium dendrite growth and mitigate interfacial issues. Typically used in small amounts (≤5–10 wt% or vol%), additives provide a cost-efficient and scalable approach to enhance AFLMB performance. Various organic and inorganic additives have been studied to regulate Li deposition, stabilize SEI formation, and improve electrochemical stability. These additives work by tuning solvation structure, forming robust interphases, and reducing side reactions at the Li/electrolyte interface. In AFLMBs, additives are especially important because they directly affect Coulombic efficiency and interfacial reversibility without excess lithium. However, many additives are rapidly consumed during cycling, limiting long-term effectiveness.^69,120,121^ Therefore, designing stable and multifunctional additives that can sustain interfacial stability and regulate Li deposition remains essential for practical applications.

**Fig. 9:**
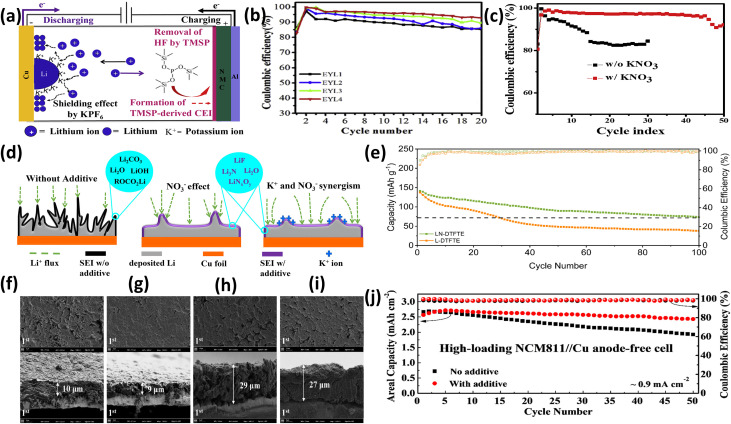
(a) Schematic illustration of the synergistic mechanism of dual additives (KPF_6_ and TMSP) in AFLMBs; (b) electrochemical performance comparison of anode-free Cu‖NMC cells in EYL1, EYL2, EYL3, and EYL4 electrolytes at 0.2 mA cm^−2^, reproduced with permission from ref. 68 (Elsevier, © 2019); (c) cycling performance of anode-free Cu//NMC cells with and without 0.5 M KNO_3_ additive cycled at 0.2 mA cm^−2^ (1st and 30th cycles); and (d) schematic illustration of the influence of KNO_3_ on lithium deposition behavior in AFLMBs; reproduced with permission from ref. 122 (Elsevier, © 2019); (e) cycling performance of LFP/Cu pouch cells in LN-DTFTE and L-DTFTE electrolytes, reproduced with permission from ref. 123 (Elsevier, © 2023); (f–i) surface and cross-sectional SEM images of Li plated on Cu foil in different electrolytes: (f) 1 mAh cm^−2^ Li plated in 1 M LDH electrolyte, (g) 1 mAh cm^−2^ Li plated in 1 M LDH + DFB electrolyte, (h) 3 mAh cm^−2^ Li plated in 1 M LDH electrolyte, (i) 3 mAh cm^−2^ Li plated in 1 M LDH + DFB electrolyte; and (j) electrochemical performance comparison of anode-free NCM811‖NMC cells with LDH and LDH + DFB electrolytes, reproduced with permission from ref. 69 (Elsevier, © 2023).

T.M. Hagos *et al.*^68^ demonstrated a dual-additive strategy using KPF_6_ and TMSP in a baseline electrolyte (1 M LiPF_6_ in EC/DEC). The optimized formulation (EYL4) significantly improved Cu‖NMC cell stability (Fig. 9a), increasing Coulombic efficiency (CE) from 89.13% to 95.21% after 20 cycles (Fig. 9b) and suppressing dendrite growth, as confirmed by more uniform Li morphology. Mechanistically, TMSP forms a protective cathode interphase and scavenges HF, while KPF_6_ enables electrostatic shielding to regulate Li deposition.

N.A. Sahalie *et al.*^122^ reported that KNO_3_ additives markedly enhance cycling stability. A 0.5 M KNO_3_ electrolyte achieved ∼41–42% capacity retention after 33–51 cycles with ∼96.6% CE, compared to rapid failure in additive-free systems (Fig. 9c). This improvement is attributed to nitrate reduction, which promotes stable SEI formation and smoother Li morphology, as evidenced by surface analysis (Fig. 9d).

K. Qin *et al.*^123^ introduced LiNO_3_ into a localized high-concentration electrolyte (LHCE), forming LiF- and Li_*x*_N_*γ*_Oz-rich SEI. This led to improved kinetics, increasing initial CE (85% → 90%) and achieving 64.5% and 52.5% capacity retention after 50 and 100 cycles, respectively (Fig. 9e). Similarly, J. Zhang *et al.*^69^ showed that LiDFOB additives enhance stability *via* cathode passivation. The optimized system delivered 94.7% capacity retention and 98.6% average CE (Fig. 9j), with more uniform and denser Li deposition (Fig. 9f–i) and reduced voltage hysteresis.

Electrolyte additive including dual-additive systems, nitrate-based SEI regulation, and borate-induced cathode passivation effectively stabilizes Li deposition, suppresses dendrites, and enhances interphase robustness. Results (Fig. 9a–j) indicate that interfacial chemistry, rather than bulk electrolyte properties, is the key factor governing Coulombic efficiency and cycling stability in AFLMBs.

#### 3.2.2 Effect of salt type on AFLMB

Advanced electrolyte systems, including high-concentration electrolytes (HCEs), localized high-concentration electrolytes (LHCEs), and weakly solvating electrolytes (WSEs), effectively improve SEI quality, Li deposition morphology, and battery lifespan by regulating the Li^+^ solvation structure. This regulation promotes preferential salt decomposition and the formation of inorganic-rich, LiF-dominated SEIs with enhanced mechanical strength and interfacial stability. The lithium salt is central to electrolyte performance, as it serves as the primary Li^+^ source and strongly influences solvation chemistry, ionic conductivity, and interphase formation. Among various salts, LiPF_6_ remains the most widely used due to its high solubility in carbonate solvents and ability to form stable, conductive electrolytes. However, its effectiveness depends on the overall electrolyte environment, underscoring the importance of coordinated salt–solvent design for improving AFLMB stability and performance.^117,124^

**Fig. 10:**
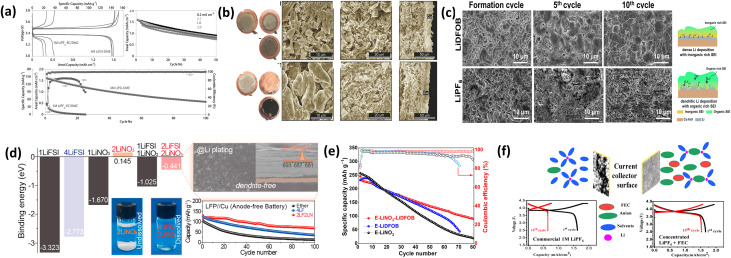
(a) Electrochemical performance of anode-free Cu‖LiFePO 4 cells with either 1 M LiPF_6_-EC/DMC or 4 M LiFSI-DME; (b) optical and SEM images of the Li plating morphology and residues remaining after discharge of first cycle plating, first cycle stripping, and 50th cycle stripping, respectively, with the 1 M LiPF_6_-EC/DMC and 4 M LiFSI-DME electrolyte, reproduced with permission from ref. ^77^ (Wiley, © 2016); (c) SEM images of plated Li on Cu foil after fully charged at different cycles with LiDFOB salt and LiPF_6_ salt, the cells are cycled from 2.8–4.5 V with a current density of 50 mA g^−1^, reproduced from ref. 125 (RSC, © 2023, CC BY 4.0); (d) calculated binding energies of the LiFSI and LiNO_3_ salts with DME as a function of salt concentration, digital photographs of different electrolytes containing LiFSI and LiNO_3_ salts in DME, SEM visualization of Cu substrates after the 1st Li plating in 2LF2LN electrolytes at 0.5 mA cm^−2^, and cycling performance and corresponding voltage profiles of the LFP/Cu AFBs using ether, 4LF, and 2LF2LN electrolytes, reproduced with permission from ref. 126 (ACS, © 2019); (e) galvanostatic cyclability of high-voltage AFLMB at 0.5C, and the corresponding charge/discharge profiles with E-LiNO_3_-LiDFOB, E-LiDFOB, and E-LiNO_3_ reproduced from ref. 67 (MDPI, © 2024, CC BY 4.0); and (f) electrolytes, the electrochemical performance of anode-free Cu‖NMC with 1 M LiPF_6_ and concentrated 2 M LiPF_6_ in EC: DEC (1 : 1, v/v) diluted with FEC reproduced with permission from ref. 127 (ACS, © 2019).

J. Qian *et al.*^77^ demonstrated that a highly concentrated ether-based electrolyte (4 M LiFSI in DME) significantly outperforms a conventional carbonate electrolyte (1 M LiPF_6_ in EC/DMC). While both systems deliver similar initial Li extraction, the carbonate electrolyte suffers from severe parasitic reactions and only ∼25% Li recovery, whereas the concentrated LiFSI electrolyte achieves a high initial Coulombic efficiency (96.6%) and stable cycling (∼60% retention after 50 cycles) with dense, dendrite-free Li deposition (Fig. 10a and b).

N.H. Hawari *et al.*^125^ compared LiDFOB and LiPF_6_ electrolytes in high-loading cells. LiDFOB forms an inorganic-rich SEI, enabling dense Li deposition, 52% capacity retention, and ∼98% CE after 50 cycles, whereas LiPF_6_ leads to organic-rich SEI, dendritic morphology, and rapid failure (Fig. 10c). The result supports the use of LiDFOB as an additive by J. Zhang *et al.*.^69^ D.W. Kang *et al.*^126^ showed that dual-salt systems (2 M LiFSI + 2 M LiNO_3_) outperform single-salt electrolytes. The optimized system achieved stable cycling (up to ∼52.7% retention) and dendrite-free Li deposition, with Li_2_O- and Li_3_N-rich SEI improving interphase stability (Fig. 10d).

Deng *et al.*^67^ further demonstrated synergistic effects of multi-salt systems. A combined LiTFSI–LiDFOB–LiNO_3_ electrolyte enabled ∼90 mAh g^−1^ retention after 80 cycles, where LiNO_3_ and LiDFOB cooperatively form Li_3_N/LiF-rich SEI and F/B-rich CEI, suppressing cathode degradation and improving reversibility (Fig. 10e). T.T. Hagos *et al.*^127^ reported a locally concentrated electrolyte (2 M LiPF_6_ in EC/DEC with FEC). The optimized system achieved 40% capacity retention and 97.8% CE after 50 cycles with uniform Li deposition and anion-derived inorganic SEI, outperforming dilute electrolytes (Fig. 10f). Salt chemistry critically governs solvation structure, SEI composition, and Li morphology. High-concentration, dual-salt, and locally concentrated electrolytes consistently promote inorganic-rich interphases (LiF, Li_3_N, Li_2_O), leading to dense Li deposition, suppressed dendrites, higher Coulombic efficiency, and improved cycling stability in AFLMBs.

#### 3.2.3 The impact of solvent type on AFLMB performance and stability

The choice of solvent, together with lithium salt and overall electrolyte composition, strongly influences Coulombic efficiency, operating voltage, temperature tolerance, and cycle life. Solvents also dominate electrolyte cost, accounting for ∼85% of total electrolyte expenses (∼30% of LIB cost), highlighting their practical significance. An ideal electrolyte solvent should exhibit a low freezing point for fluidity, an appropriate dielectric constant for high ionic conductivity, a suitable donor number (DN) for effective salt solvation, and low viscosity for rapid ion transport. These properties must be carefully balanced, as they directly affect solvation structure, interfacial reactions, and overall AFLMB stability.^128^

**Fig. 11:**
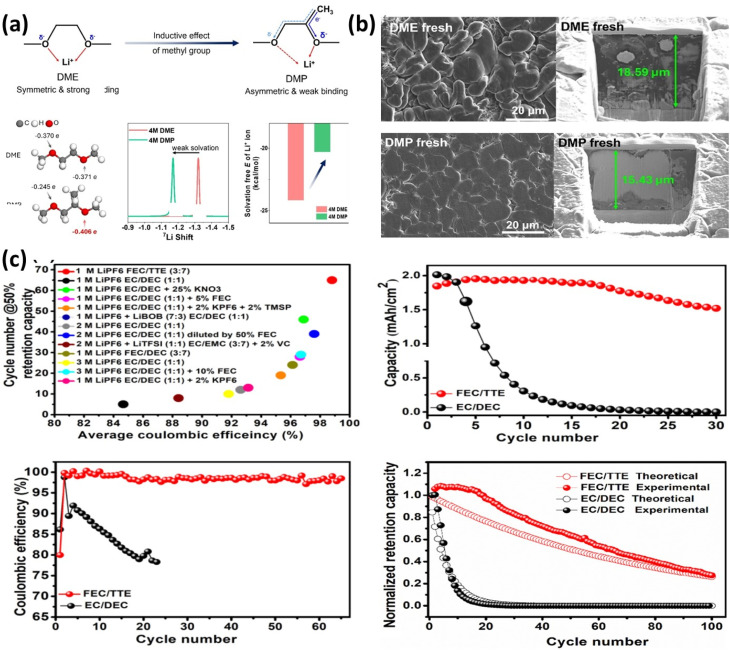
(a) Illustration of the inductive effect of electrolytes, atomic charges of oxygen sites in DME and DMP molecules, ^7^Li NMR spectra of 4 M DME and 4 M DMP, and solvation free energies of Li^+^ in 4 M DME and 4 M DMP; (b) morphology of Li deposits after the 5th plating at 0.4 mA cm^−2^ and 4 mAh cm^−2^: top-view (left) and cross-sectional view (right) in 4 M DME and 4 M DMP without rest, reproduced with permission from ref. 37 (Elsevier, © 2024); (c) electrolyte performance evaluation in Cu//NMC anode-free cells cycled at 2.5–4.5 V (0.5 mA cm^−2^): retention capacity at 50% *vs.* average CE and cycling stability comparison between 1 M LiPF6 in FEC/TTE (3 : 7 v/v) and 1 M LiPF6 in EC/DEC (1 : 1 v/v), reproduced with permission from ref. 129 (Elsevier, © 2020).

Lee *et al.*^37^ investigated 1,2-dimethoxypropane (DMP) as a weakly solvating solvent in 4 M LiFSI and compared it with DME. The weaker solvation in DMP (confirmed by ^7^Li NMR, Fig. 11a) promotes inorganic-rich (Li_2_O-rich) SEI formation, leading to dense and uniform Li deposition with reduced thickness and corrosion (Fig. 11b). As a result, AFLMBs with DMP showed more stable capacity retention (51% → 49%) compared to DME (47% → 32%). This demonstrates that weakly solvating solvents effectively suppress Li loss and stabilize interphases.

T.T. Hagos *et al.*^129^ developed a carbonate–ether mixed solvent (FEC/TTE) with LiPF_6_ for high-voltage AFLMBs. Compared to conventional EC/DEC electrolytes with rapid failure, the optimized FEC/TTE system achieved ∼85% capacity retention at 30 cycles and >50% after 65 cycles with ∼98.7% CE (Fig. 11c). XPS analysis confirmed a fluorine-rich SEI, while stable cycling indicated robust interphase formation. The improved performance arises from synergistic effects of solvated ion pairs, LiF-rich SEI, and CEI formation, which suppress interfacial degradation. Solvent selection critically controls solvation structure, SEI chemistry, and Li morphology; weakly solvating and fluorinated/mixed solvents promote stable, inorganic-rich interphases, enabling reduced corrosion, suppressed side reactions, and improved cycling stability in AFLMBs.

**Fig. 12:**
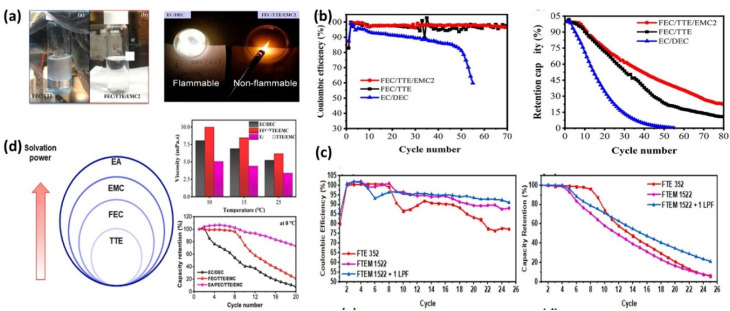
(a) Digital images of prepared electrolytes and flammability test of EC/DEC and FEC/TTE/EMC2, and FEC/TTE (3 : 7 by vol.) in 1 M LiPF_6_; (b) comparison of the electrochemical performance (Retention capacity and CE) using Cu‖NMC111 anode-free cells within of EC/DEC, FEC/TTE, and FEC/TTE/EMC2 electrolytes at current density of 0.5 mA cm^−2^, reproduced with permission from ref. 130 (ACS, © 2020); (c) CE, and capacity retention comparison of FTE 352, FTEM 1522 and FTEM 1522 + 1 LPF in Cu‖NMC111 at 0 °C within 2.5–4.5 V using 0.2 mA cm^−2^ current density, reproduced with permission from ref. 131 (Elsevier, © 2022); and (d) comparison of solvation power, viscosity, and low-temperature cycling performance using Cu‖NMC111 anode-free cells of EC/DEC, FEC/TTE/EMC, and EA/FEC/TTE/EMC within the voltage range of 2.5–4.5 V at current densities of 0.2 mA cm^−2^, reproduced with permission from ref. 132 (Elsevier, © 2021).

Hagos *et al.*^130^ improved the FEC/TTE electrolyte by introducing EMC, forming 1 M LiPF_6_ in FEC/TTE/EMC (3 : 5 : 2). This modification eliminated phase instability and reduced viscosity while enhancing ionic conductivity and oxidative stability (Fig. 12a). The optimized system achieved ∼40% capacity retention and 98.30% CE over 80 cycles in Cu‖NMC111 cells (Fig. 12b), with compact, nodular Li deposition suppressing dendrite formation. The Hwang group^131^ further introduced a quaternary system (FEC/TTE/EMC/MA) with LiPO_2_F_2_ additive. This electrolyte improved low-temperature performance (96% CE at 0 °C *vs.* 94.4% for the ternary system) and enhanced rate capability, achieving 48% capacity retention at 2 mA cm^−2^ (Fig. 12c). The improvements arise from reduced viscosity (*via* MA) and dendrite suppression (*via* LiPO_2_F_2_). T.M. Hagos *et al.*^132^ subsequently incorporated EA into FEC/TTE/EMC to enable wide-temperature operation. The EA-containing electrolyte exhibited lower viscosity and higher ionic conductivity (Fig. 12d), delivering 73% capacity retention and 95% CE at 0 °C, significantly outperforming baseline systems. Enhanced performance is attributed to improved ionic mobility, despite a slightly less inorganic-rich SEI. Solvent engineering through fluorinated, multi-component, and low-viscosity systems effectively tunes solvation structure, reduces viscosity, and stabilizes interphases, enabling improved cycling stability, dendrite suppression, and wide-temperature performance in AFLMBs.

#### 3.2.4 Composite-based electrolyte for AFLMBs

Replacing flammable liquid electrolytes with all-solid-state systems (inorganic, polymeric, or hybrid) is promising for AFLMBs; however, their effectiveness depends on addressing interfacial instability, dendrite growth, and uneven lithium deposition. Solid polymer electrolytes (SPEs) improve interfacial compatibility with the Cu current collector by accommodating surface roughness, reducing localized current density and lithium nucleation heterogeneity. While they can help suppress dendrite growth, dendrite control depends on interfacial stability and ion transport rather than mechanical strength alone. Their performance is limited by ionic conductivity and interfacial resistance, so their advantages must be critically evaluated based on failure mechanisms and operating conditions.^124,133,134^

**Fig. 13:**
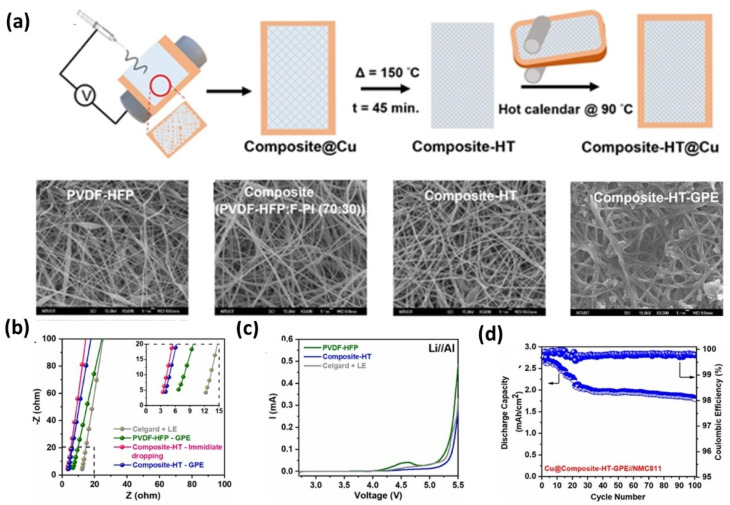
(a) Schematic of the preparation of the Composite-HT polymer membrane and corresponding SEM images (×3000) of the electrospun nanofibrous membranes: PVDF-HFP, composite (PVDF-HFP : F-PI, 70 : 30), heat-treated composite (Composite-HT), and Composite-HT after gelation with liquid electrolyte (1.5 M LiFSI + 0.058 M LiDFOB in DME : TTE, 1 : 4 v/v), forming the Composite-HT-GPE; (b) EIS plots (SUS//SUS) comparing Celgard + LE, PVDF-HFP, and Composite-HT polymer matrices before and after gelation; (c) linear sweep voltammograms (Li//Al) of PVDF-HFP, Composite-HT-GPE, and Celgard + LE; (d) cycling performance of pouch cells with Cu@Composite-HT-GPE//NMC811, reproduced with permission from ref. 135 (Elsevier, © 2024).

T. Agnihotri *et al.* developed an anion-trapping gel polymer electrolyte (GPE) based on a composite of Poly(vinylidene fluoride-*co*-hexafluoropropylene) (PVDF-HFP) and Fluorinated Polyimide (F-PI).^135^ The composite films were gelled in a LiFSI/LiDFOB-based solvent system, yielding an optimized GPE with enhanced structural stability (Fig. 13a). The hydrogen–bonding interaction between PVDF-HFP and F-PI not only improves mechanical integrity but also enhances Li^+^ transport pathways, indicating a coupled role of polymer chemistry and ion conduction rather than relying on a single mechanism (Fig. 13b). Electrochemical analysis showed an expanded stability window from 4.2 to 5.0 V (Fig. 13c), suggesting suitability for high-voltage cathodes; however, such stability must be interpreted in conjunction with interfacial kinetics and long-term compatibility rather than voltage window alone. In anode-free cells, the electrolyte delivered ∼70% capacity retention and 99.8% CE after 100 cycles (Fig. 13d), with SEM confirming relatively uniform lithium deposition. Importantly, these results highlight improved interfacial stability, but performance degradation over extended cycling still indicates unresolved interfacial and transport limitations. Overall, this system illustrates how anion-trapping and interfacial engineering can partially address dendrite suppression and stability issues, but further optimization is required for practical high-current operation.

**Fig. 14:**
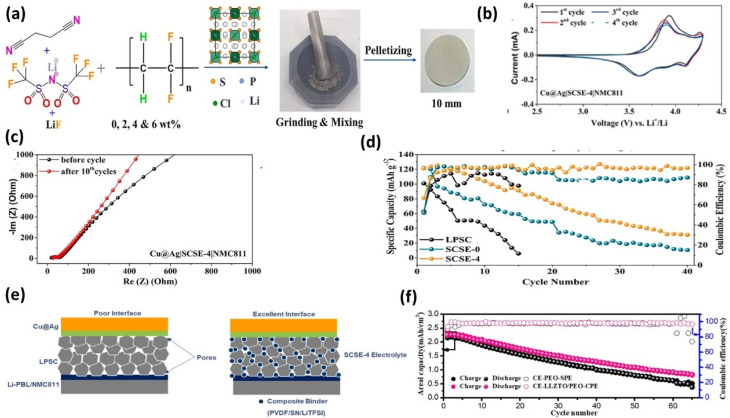
(a) Schematic of SCSE-*x* preparation (*x* = 0, 2, 4, 6 wt% PVDF); (b) cycling performance of Cu@Ag‖NMC811 cells with LPSC, SCSE-0, and SCSE-4 electrolytes; (c) EIS spectra of Cu@Ag|SCSE-4|NMC811 before and after 10 cycles; (d) CV curves of Cu@Ag|SCSE-4|NMC811 scanned at 0.2 mV s^−1^; (e) schematic of ultrathin laminated LLZTO/PEO-CPE on anode and cathode surfaces, reproduced with permission from ref. 136 (Elsevier, © 2024); (f) cycling performance comparison of Cu‖PEO-SPE‖NMC and Cu‖LLZTO/PEO-CPE‖NMC cells at 0.2 mA cm^−2^ and 55 °C, reproduced with permission from ref. 78 (ACS, © 2020).

N.T. Temesgen *et al.* proposed a solvent-free sulfide composite solid electrolyte (SCSE-4) by incorporating Li_6_PS_5_Cl into a SN–LiTFSI matrix with PVDF and LiF additives.^136^ This design primarily targets interfacial stability and mechanical deformability, linking directly to failure mechanisms such as interfacial resistance and contact loss. Among the formulations, SCSE-4 exhibited superior electrochemical performance, maintaining higher discharge capacity and CE compared to LPSC and SCSE-0, even under higher current density. The improved performance can be attributed to reduced interfacial resistance and enhanced Li^+^ transport, rather than compositional complexity alone. The observed A/C > 1 behavior suggests progressive activation of electrochemically accessible lithium, which indicates evolving interfacial dynamics rather than purely stable cycling. EIS and CV analyses confirmed improved kinetics and reversibility (Fig. 14b–e), although these results are limited to moderate current densities and require validation under more demanding conditions. Overall, this work demonstrates that composite design can address multiple failure mechanisms simultaneously, but clear benchmarking against other electrolyte systems remains necessary.

T.A. Zegeye *et al.* developed a garnet–polymer composite electrolyte (LLZTO/PEO-CPE) laminated on both electrodes.^78^ The composite exhibits high ionic conductivity (∼4.76 × 10^−4^ S cm^−1^) and reduced interfacial resistance, enabling stable Li plating. Anode-free cells achieved an average CE of 98.8% and 41.2% capacity retention after 65 cycles at 55 °C (Fig. 14f). While these results indicate improved interfacial stability and dendrite suppression, the performance remains strongly temperature-dependent and limited in long-term cycling stability. Moreover, the relatively modest capacity retention highlights that achieving high CE alone is insufficient for practical deployment, emphasizing the need for balanced optimization of conductivity, stability, and mechanical properties. Overall, this strategy demonstrates that integrated electrode–electrolyte design is critical for mitigating failure mechanisms, but further work is needed to establish scalability.

### 3.3 Cycling protocol design for AFLMBs

Cycling protocols are critical for AFLMB performance, alongside interfacial and electrolyte design; however, their role must be understood in relation to specific failure mechanisms rather than as general performance enhancers. Key parameters including formation protocols, voltage window, current density, temperature, and pressure govern SEI formation, lithium deposition behavior, and interfacial stability.^137^ Specifically, formation conditions influence SEI stability, current density affects dendrite growth, and pressure/temperature regulate interfacial contact and dead lithium formation. While optimized protocols can improve cycling stability, their effectiveness remains condition-dependent and requires systematic comparison across studies. Therefore, a clear linkage between cycling parameters and failure mechanisms is essential for identifying effective strategies.^75,138,139^ The following sections analyze formation rate, temperature, pressure, and nucleation control from this perspective.

#### 3.3.1 Effect of formation rate on the stability of AFLMBs

Cell formation is a critical process governing Li nucleation and growth in AFLMBs. Formation rate directly influences failure mechanisms, where high current densities (10–30 mA cm^−2^) can promote ultrafine, uniformly distributed Li nuclei on the Cu current collector, improving deposition uniformity and suppressing dendrite growth. This behavior is attributed to increased nucleation density and modified SEI chemistry, rather than current alone. High-current formation also favors the formation of inorganic-rich SEI *via* enhanced anion decomposition, which can improve interfacial stability and charge transfer.^140,141^ However, these benefits are condition-dependent and may not be sustained under practical cycling conditions.

**Fig. 15:**
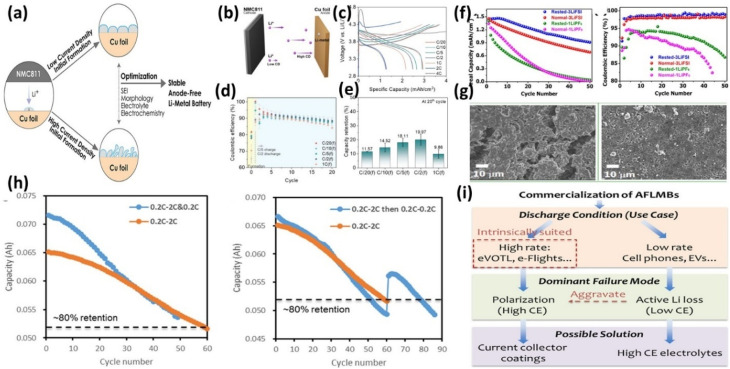
(a) Schematic representation of the effect of CD on lithium metal deposition; (b) Schematic of a Cu‖NMC811 AFB system; (c) initial cycle voltage profiles of Cu‖NMC811 AFBs with various initial charging CDs and a fixed discharging CD at *C*/2; (d) Coulombic efficiency of AFBs, reproduced from ref. 80 (ACS, 2016, CC BY 4.0); (e) capacity retention after the 20th cycle of AFBs; (f) comparison of discharge capacity and CE profiles of cells in Rested-1LiPF_6_, Normal-3LiFSI, Rested-3LiFSI, and Normal-1LiPF_6_; (g) SEM morphological evaluation of Normal-3LiFSI and Rested-3LiFSI after 100 cycles, reproduced with permission from ref. 72; (ACS, © 2019), (h) cycling results after applying protocol #1 and applying protocol #2; and (i) strategies to improve AFLMB cycle life under different use cases, reproduced with permission from ref. 45 (ACS, © 2023).

S. Kim *et al.*^80^ showed that formation current density (CD) strongly affects cycling stability in NMC811‖Cu AFLMBs (Fig. 15a–e). While high CDs (≥2*C*) increased overpotential and induced electrolyte decomposition (>4.3 V) (Fig. 15c), moderate formation rates (*e.g.*, *C*/2) achieved optimal performance by balancing Li nucleation and SEI formation, leading to improved capacity retention compared to *C*/20 (Fig. 15e). This indicates that formation CD governs dendrite suppression and SEI evolution, rather than simply enhancing performance with increasing current density.

Beyene *et al.*^72^ demonstrated that a controlled formation protocol with a resting step enables the formation of a stable F-rich SEI, mitigating dendrite growth and improving interfacial stability, resulting in high CE and extended cycling (Fig. 15f and g). This highlights the importance of temporal control (resting) in addition to current density.

Wang *et al.*^45^ found that discharge rate primarily influences polarization and Li utilization rather than direct Li loss. A high-rate discharge protocol improved cycle life (Fig. 15h), indicating that capacity decay is dominated by kinetic limitations, while high charge rates reduced long-term CE (Fig. 15i), revealing a trade-off between kinetics and stability. Overall, these results show that cycling protocols must be explicitly linked to failure mechanisms (SEI instability, dendrite growth, polarization), and their effectiveness depends on electrolyte chemistry and operating conditions rather than a single optimized parameter.

#### 3.3.2 Effect of the pressure on the stability of AFLMBs

Applying stacking pressure is an effective strategy to regulate lithium deposition and reduce dead lithium formation in AFLMBs; however, its impact must be understood in relation to specific failure mechanisms rather than as a universally beneficial parameter. In particular, pressure governs the chemo-mechanical environment at the electrode interface, directly affecting Li nucleation, growth uniformity, and interfacial contact stability. Pressure primarily influences interfacial contact, lithium morphology, and dead lithium evolution, with limited direct impact on SEI formation.^142^

**Fig. 16:**
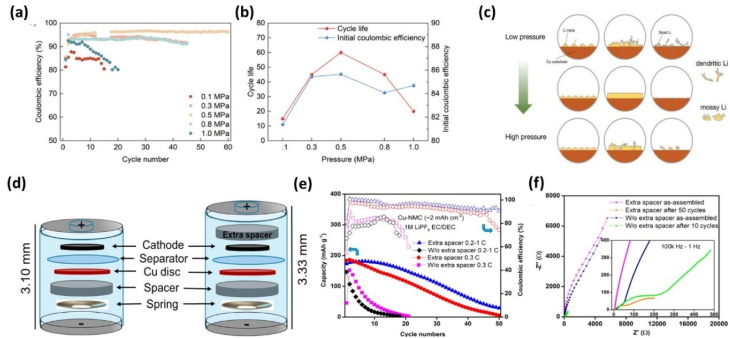
(a) CE over the full life of AFLMBs at different stacking pressures; (b) trends of initial CE and battery life with stacking pressure; (c) schematic representation of the effect of stacking pressure on lithium metal deposition and dead lithium formation, reproduced with permission from ref. 81 (Wiley, © 2024); (d) the normal configuration of an anode-free Li-metal battery in a coin cell 2032 and with an extra spacer at the cathode side; (e) capacity retention and CE of AFLMBs using NMC cathode; and (f) Nyquist plots of the anode-free Li metal batteries charged at 0.2C and discharged at 1C before/after cycles, with/without an extra spacer reproduced from ref. 76 (ECS, 2021, CC BY 4.0).

Lin *et al.*^81^ showed that increasing pressure from 0.1 to 0.5 MPa reduced polarization and improved cycling stability (CE ∼96%), with optimal performance at 0.5 MPa (Fig. 16a–c). This improvement arises from enhanced interfacial contact and more uniform Li deposition, which lowers local current density. Low pressure led to dendritic Li, while excessive pressure induced cracking and mossy Li, indicating distinct failure pathways. These observations suggest that both insufficient mechanical constraint and excessive stress can destabilize Li morphology through different mechanisms. Notably, dead lithium formation was minimized at intermediate pressure, highlighting the importance of balancing mechanical constraint and structural integrity.

Zhou *et al.*^76^ further demonstrated that applying pressure *via* spacers improves interfacial contact and reduces resistance, leading to enhanced CE (>90%) and cycle life (Fig. 16d–f). The spacer effectively maintains electrode alignment during cycling, mitigating contact loss and suppressing localized current hotspots. This confirms that pressure mainly mitigates contact loss and dead lithium accumulation rather than fundamentally altering SEI chemistry.

**Fig. 17:**
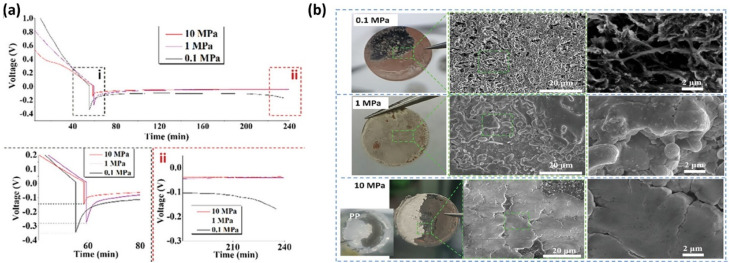
(a) Voltage profiles of Li plated onto Cu‖Li at 1 mA cm^−1^, with external pressure of 10, 1, or 0.1 MPa. Subpanels (i) and (ii) show expanded portions of the nucleation and growth sections, respectively; and (b) low magnification optical light microscope images and high magnification SEM images of the Li metal surface with capacity of 3 mAh cm^−2^ at 1 mA cm^−2^. at 10, 1, and 0.1 MPa, respectively, reproduced with permission from ref. 143 (Wiley, © 2023).

Liu *et al.*^143^ investigated the effect of external pressure (0.1–10 MPa) on SEI structure and Li plating behavior in Cu‖Li cells. Increasing pressure reduced nucleation overpotential from 340 to 150 mV (Fig. 17a), indicating lower kinetic barriers for Li nucleation due to chemo-mechanical effects. At low pressure (0.1 MPa), the overpotential increased during growth, whereas at higher pressures (1–10 MPa) it remained stable (∼45 mV), suggesting more uniform deposition. Higher pressure also increased the density and coverage of Li nuclei, promoting homogeneous growth. These trends are consistent with Lin *et al.*^81^ and Zhou *et al.*,^76^ and EIS results (Table 3) further confirm reduced interfacial resistance at higher pressures.

**Table 3:** EIS fitting results for Li‖Cu cells after lithium plating under different external pressures.^143^

Impedance [Ω]	*R*_Ohm_	*R*_SEI−1_	*R*_SEI−2_	*R*_SEI−1_ + *R*_SEI−2_	*R*_ct_
0.1 MPa (lithiated)	6.1	3.9	23.5	27.4	13.9
0.1 MPa (plated)	14.8	3.9	28.5	32.4	11.9
1 MPa (lithiated)	2.8	6.2	23.8	30	12.4
1 MPa (plated)	5.8	6.1	29.9	36	12.5
10 MPa (lithiated)	2.7	5.2	18.2	23.4	10.6
10 MPa (plated)	2.7	4.9	19.0	23.9	10.6

Table 3 further indicate reduced interfacial resistance at higher pressures. The EIS results show a clear pressure-dependent reduction in interfacial resistance. Increasing pressure from 0.1 to 10 MPa lowers *R*_ohm_, indicating improved electrode contact, while high SEI resistance at 0.1 MPa reflects a porous, unstable interphase associated with dead lithium formation. Moderate pressure (1 MPa) improves contact and stabilizes the interface, whereas higher pressure (10 MPa) further reduces SEI and charge-transfer resistance, suggesting enhanced Li^+^ transport and more uniform deposition. However, the limited improvement at high pressure indicates diminishing electrochemical benefits, highlighting that an optimal pressure is required to balance interfacial stability and mechanical integrity.

SEM analysis (Fig. 17b) shows that Li morphology strongly depends on applied pressure. At 0.1 MPa, deposits are porous and uneven, with micron-scale voids and limited Cu coverage, indicating heterogeneous, dendritic growth. At 1 MPa, deposits become more compact with reduced porosity and improved surface coverage. At 10 MPa, the film is highly uniform with low roughness and minimal porosity, reflecting dense and homogeneous Li deposition. However, excessive pressure can damage cell components, as evidenced by PP separator rupture at 10 MPa, highlighting a critical limitation. Overall, low pressure favors porous, filament-like Li with unstable SEI, whereas high pressure promotes dense Li and more inorganic, F-rich SEI.

#### 3.3.3 Effect of the temperature on the stability of AFLMBs

**Fig. 18:**
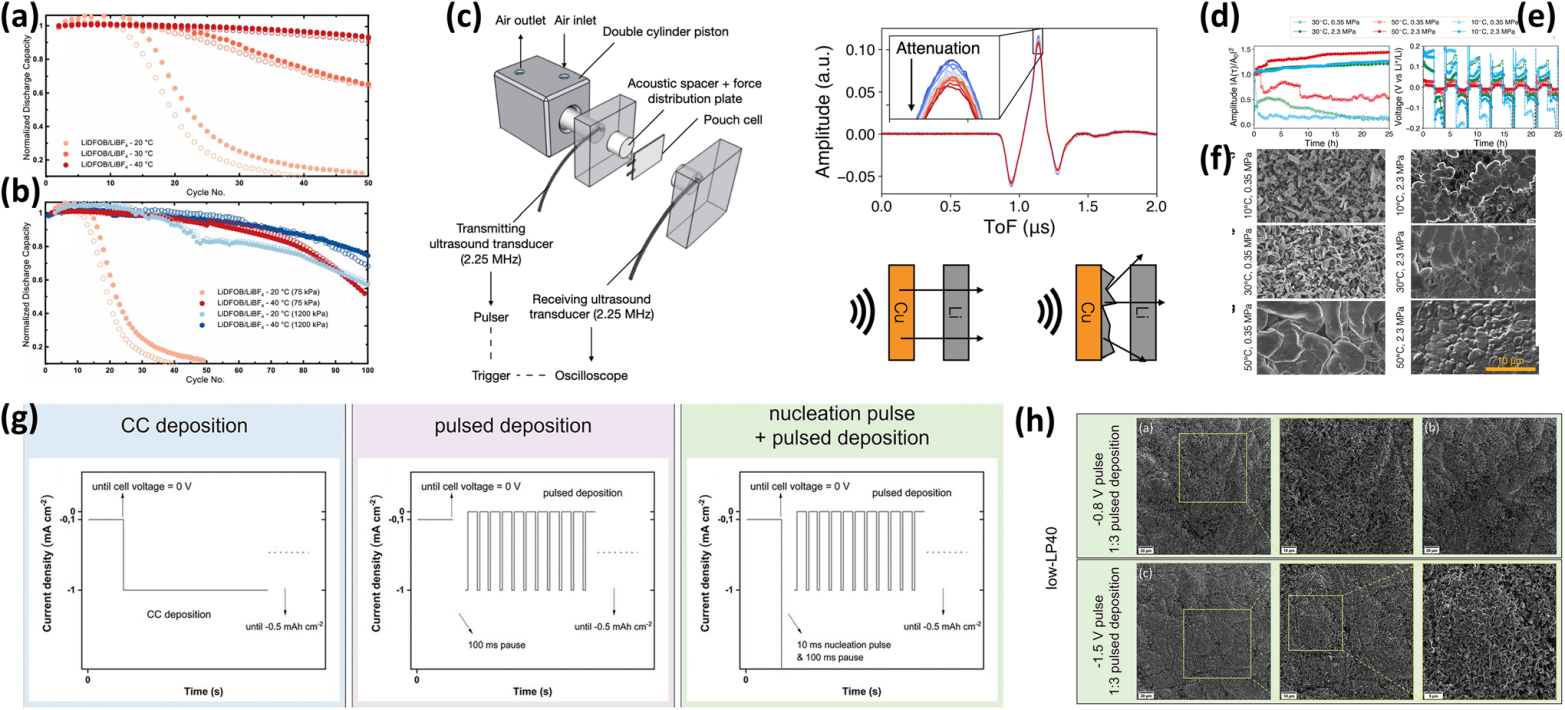
(a) Normalized discharge capacity *vs.* cycle number for NMC532‖Cu anode-free cells with 0.6 M LiDFOB + 0.6 M LiBF_4_ in FEC : DEC (1 : 2) electrolyte cycled at 20 °C, 30 °C, and 40 °C; (b) comparison of discharge capacity retention under low (20 °C) and high (40 °C) temperature conditions and low (75 kPa) *vs.* high (1200 kPa) stack pressure, reproduced with permission from ref. 74 (ECS, © 2019, CC BY 4.0); (c) schematic of a constant-pressure holder for *operando* acoustic transmission tests and example waveforms showing amplitude attenuation caused by non-uniform Li deposition; (d) acoustic amplitude over 10 plating/stripping cycles in Li‖Cu pouch cells; (e) voltage profiles corresponding to the plating and stripping cycles; (f) SEM images of Li deposition on Cu after 11 plating steps under different temperatures and stack pressures, reproduced with permission from ref. 73 (ECS, © 2022, CC BY 4.0); (g) schematic of protocols used in Li electrodeposition experiments; (h) SEM images showing Li morphologies on prelithiated Cu substrates in low-LP40 electrolyte with different potentiostatic nucleation pulse heights, reproduced with permission from ref. 34 (Wiley, © 2023, CC BY 4.0).

Temperature plays a critical but non-linear role in AFLMB stability and is inherently coupled with stack pressure, electrolyte properties, and formation protocols rather than acting as an isolated variable.^144,145^ At low temperatures, reduced ionic conductivity and sluggish kinetics lead to increased overpotentials, non-uniform lithium nucleation, and subsequent dead lithium formation and SEI degradation. These coupled effects are identified as the primary failure mechanisms, providing a clearer mechanistic interpretation. Genovese *et al.*^74^ (Fig. 18a and b) showed that hot formation at 40 °C enables over 80% capacity retention after 50 cycles, whereas lowering temperature to 30 °C and 20 °C reduces stability to ∼30 and ∼18 cycles to 80% retention, respectively.

Stack pressure plays a synergistic role in mitigating low-temperature degradation by improving interfacial contact and suppressing voids, extending cycle life at 20 °C from ∼18 to ∼50 cycles to 80% retention at 1200 kPa. However, at 40 °C, the marginal improvement suggests that kinetic limitations are no longer dominant, and interfacial or chemical stability becomes the limiting factor. Hot formation (40 °C) followed by low-temperature cycling (20 °C) improves performance (∼60 cycles to 80% retention), and when combined with high pressure, achieves ∼85% retention after 100 cycles. This strengthens the direct linkage between failure mechanisms and mitigation strategies. Chang *et al.*^73^ (Fig. 18c–f) further confirm that low temperature and pressure cause high acoustic attenuation, poor contact, and non-uniform lithium growth, whereas increasing both parameters improve morphology and reduces overpotentials, with lithium plastic deformation under pressure acting as a key stabilization mechanism.

AFLMB stability arises from the synergistic optimization of temperature, pressure, and formation protocols, each addressing distinct failure modes such as kinetic limitation, interfacial instability, and morphological inhomogeneity. As elevated temperature, while beneficial for kinetics, is constrained by electrolyte decomposition, SEI instability, and safety concerns. A key limitation in current studies is the lack of clear mapping between failure mechanisms and control strategies, which limits cross-study comparison.

#### 3.3.4 Effect of the potentiostatic nucleation pulse on the stability of AFLMBs

Among various electrochemical control strategies, nucleation regulation *via* transient potential control offers a direct route to influence the initial Li deposition pathway, which ultimately dictates long-term cycling stability.

Chen *et al.*^34^ studied the effect of a potentiostatic nucleation pulse on Li deposition using Cu‖Li three-electrode cells with LP40 electrolyte. After preconditioning, Li was deposited *via* constant current, pulsed current, or pulsed current with a nucleation pulse (Fig. 18g). A high-overpotential pulse increases Li nuclei density, promoting uniform 2D growth over uneven 3D deposition. This is particularly important for Cu substrates with abundant grain boundaries that induce heterogeneous nucleation. As shown in Fig. 18h, this approach suppresses localized Li buildup, directly mitigating dendrite initiation and dead Li formation, although it does not address SEI chemical instability. Nucleation pulses of −0.8 V and −1.5 V produced distinct morphologies (Fig. 18h), with −1.5 V yielding more compact and homogeneous deposits. The low-LP40 electrolyte showed greater improvement due to higher effective overpotential. The result demonstrate that nucleation pulse control is a simple and effective strategy for improving early-stage deposition uniformity, but its impact must be coupled with electrolyte and SEI optimization for long-term stability.

## 4. Degradation mechanisms and advanced characterization tools in AFLMBs

### 4.1 Degradation mechanisms in AFLMBs

AFLMBs operate without pre-deposited lithium on the anode; instead, lithium is reversibly plated/stripped on a bare current collector (typically Cu). While this design enables high energy density, its practical viability is severely constrained by the absence of lithium excess, meaning even minimal irreversible lithium loss leads to rapid failure. Thus, degradation in AFLMBs must be evaluated not only by type but by severity, coupling, and impact on Coulombic efficiency under realistic conditions. To address this, Table 4 correlates failure mechanisms with their causes and targeted mitigation strategies, enabling a more application-relevant interpretation.

**Table 4:** Degradation of mechanisms and its main causes in AFLMBs

Mechanism	Main cause	Effect on cell	Mitigation strategies	Key references
Dendrite growth	Uneven Li deposition due to current hotspots and high nucleation overpotential	Causes short circuit, rapid Li loss	Lithophilic coatings, controlled current, solid electrolytes	146
Dead lithium	Isolated or SEI-trapped Li after uneven stripping	Reduces active Li inventory and capacity	Smooth deposition, stable SEI, pressure control	147
SEI instability	Cracking/reforming from volume and chemical changes	Continuous Li/electrolyte loss, high impedance	F-rich electrolytes, artificial SEI layers	148
Coupled degradation cycle	Interaction of SEI failure, dendrite growth, and dead Li formation	Accelerated capacity fade, early failure	Integrated interface and electrolyte design	149
Overall impact	Small irreversible Li losses per cycle	Rapid performance decay in anode-free systems	System-level optimization of interface, electrolyte, and pressure	147

Degradation in AFLMBs is governed by three strongly coupled processes: dendrite growth, dead lithium formation, and SEI instability. Unlike conventional interpretations, these mechanisms do not act independently but evolve as a feedback loop that dictates failure progression.

Dendrite growth originates from kinetically driven non-uniform lithium nucleation, rather than purely mechanical instability.^146^ While strategies such as lithophilic coatings reduce nucleation barriers and improve deposition uniformity, commonly used approaches such as lowering current density introduce a critical trade-off with power performance, limiting their practical applicability. Currently, more advanced nucleation-control strategies have emerged. For example, the concept of confining lithium in a continuous nucleation state provides a pathway to regulate lithium plating at the atomic scale. By maintaining uniformly distributed nucleation sites and minimizing localized current density fluctuations, this approach effectively suppresses dendritic growth while simultaneously reducing the formation of electrically isolated “dead” lithium.^150^

Dead lithium formation is the primary driver of capacity fade, as electrically isolated lithium is permanently removed from cycling. Importantly, AFLMB failure is more often governed by cumulative lithium loss than by short circuit, underscoring the importance of achieving high Coulombic efficiency. Approaches such as pressure optimization and dense lithium deposition improve interfacial contact and reversibility;^147^ however, excessive pressure may induce SEI fracture and accelerate parasitic reactions, highlighting the need for balanced optimization.

SEI instability acts as the fundamental origin of continuous degradation, where repeated fracture and reformation consume lithium and electrolyte while increasing interfacial resistance. Critically, unstable SEI accelerates both dendrite growth and dead lithium formation, functioning as a central “failure amplifier”. Although fluorine-rich electrolytes and artificial SEI layers can enhance stability,^148^ their effectiveness is highly dependent on operating conditions and is often overstated under low-loading laboratory conditions.

These mechanisms are intrinsically coupled: SEI instability induces heterogeneous lithium deposition, promoting dendrite growth; dendrites increase lithium isolation, forming dead lithium; and dead lithium further amplifies current inhomogeneity, exacerbating SEI instability.^149^ This self-reinforcing degradation loop explains why single-strategy solutions often fail to deliver meaningful improvements in practical systems.

From a quantitative perspective, AFLMBs require Coulombic efficiency exceeding ∼99.9% to achieve practical cycle life, as even minimal lithium loss accumulates rapidly due to the absence of a lithium reservoir.^147^ This stringent requirement reveals a critical gap between laboratory-scale demonstrations and real-world applicability, where many reported systems operate under conditions that do not reflect practical constraints such as high areal capacity and lean electrolyte configurations.

### 4.2 Advanced characterization tools for AFLMBs

Advanced characterization techniques are essential for understanding and improving AFLMB performance, as they provide direct insight into lithium plating/stripping behavior, interfacial evolution, and degradation pathways. However, beyond simple observation, their true value lies in linking measurable signals to specific failure mechanisms such as dead lithium formation, SEI instability, and dendrite growth.

To address this, Table 5 not only summarizes key techniques but also clarifies their unique diagnostic role in resolving critical bottlenecks in AFLMBs, enabling a more targeted and mechanism-driven interpretation.

**Table 5:** Advanced characterization tools and their diagnostic roles in AFLMBs

Tools	Key strengths	What it reveals in AFLMBs	References
Synchrotron X-ray tomography (XTM)	Non-destructive 3D imaging (*operando*)	Tracks Li plating/stripping, voids, and short initiation	151
Cryogenic TEM (Cryo-TEM)	Atomic-scale imaging under cryo protection	Identifies SEI nanostructure, dendrites, and dead Li	152
PFIB-SEM tomography	Fast 3D microstructure mapping	Visualizes Li distribution, porosity, and interface evolution	153 and 154
*Operando* EIS	Real-time electrical analysis	Monitors SEI growth, resistance, and shorting signals	155
XPS	Surface chemistry identification	Determines SEI components and oxidation states	156
ToF-SIMS	Depth-resolved chemical imaging	Maps ions and organic fragments in SEI layers	157
Neutron imaging	Probes through metal casings	Visualizes Li plating inside full cells	158
Raman/FTIR	Vibrational spectroscopy	Tracks electrolyte and SEI chemical changes	57
AFM/EC-AFM	Nanoscale mechanical/topo mapping	Measures SEI roughness and elasticity	159
*Operando* XRD	Monitors phase evolution	Detects structural and alloy changes during cycling	159
Cryo-FIB-STEM	High-resolution cross-sectioning	Preserves true Li/SEI interface at atomic level	160
*In Situ* optical microscopy	Real-time visual observation	Captures dendrite nucleation and growth dynamics	161

For dendrite growth, real-time visualization tools such as *in situ* optical microscopy and synchrotron X-ray tomography directly capture nucleation and propagation behavior.^154^ However, these methods are limited in resolving early-stage nucleation at the nanoscale, where techniques such as Cryo-TEM become essential for identifying initial deposition heterogeneity and dendrite precursors.^155^

Dead lithium formation, the dominant contributor to capacity fade, is best resolved through 3D structural and connectivity analysis. PFIB-SEM tomography and X-ray tomography reveal lithium isolation, void formation, and loss of electronic pathways.^154,156^ This highlights that dead lithium is not merely a chemical issue but a structural and connectivity problem, requiring combined morphological and electrochemical diagnostics.

SEI instability, which underpins continuous degradation, requires multi-modal chemical and mechanical characterization. Techniques such as XPS and ToF-SIMS provide compositional and depth-resolved chemical information,^159,160^ while AFM/EC-AFM reveals mechanical properties such as stiffness and fracture behavior. *Operando* EIS complements these by tracking resistance evolution and interfacial instability in real time.^158^ Importantly, no single technique can fully describe SEI behavior, necessitating integrated analysis across multiple scales.

Despite their powerful capabilities, advanced characterization techniques face several limitations that constrain their practical impact. High-resolution methods such as Cryo-TEM and Cryo-FIB-STEM often require complex sample preparation and operate under non-representative conditions, potentially altering the native lithium structure.^155^ Similarly, *operando* techniques may sacrifice spatial resolution for temporal insight, leading to incomplete interpretation of interfacial phenomena. Another critical challenge is the lack of standardized testing conditions, making it difficult to directly compare results across studies. Many characterization results are obtained under simplified conditions (low current density, thin electrodes), which may not reflect realistic battery operation.

## 5 Summary and future perspectives

Anode-free lithium-metal batteries (AFLMBs) eliminate pre-deposited anodes, forming lithium *in situ* on a bare copper current collector. This architecture enhances cathode utilization, increases energy density, and reduces lithium consumption. Despite these advantages, practical deployment faces critical challenges, including dendrite growth, dead lithium formation, SEI instability, low Coulombic efficiency, and limited electrolyte stability. Recent experimental studies provide strategies to address these issues.

### 5.1 Protective interlayers and interface engineering

Ultrathin polymer or alloy coatings on copper guide uniform lithium nucleation and stabilize the SEI, improving cycle life and Coulombic efficiency (CE). These layers simultaneously mitigate dendrites and prevent SEI degradation.

### 5.2 Advanced electrolyte formulations

Donor-number (DN) modulated localized high-concentration electrolytes (LHCEs) and fluorinated solvents form inorganic-rich SEI, suppress dendrites, and achieve CE ≈ 99% over extended cycling. High-voltage stable electrolytes enable operation at 4.6 V, enhancing cathode utilization (MDPI, 2025).

### 5.3 Next-generation solid electrolytes

Polymer–ceramic composites and sulfide-based SSEs (*e.g.*, Li6PS5Cl) provide mechanical suppression of dendrites and improved safety. However, interfacial stability and long-term cycling remain bottlenecks, requiring further material and interface engineering.

### 5.4 Prelithiation techniques

Chemical (SLMP) or electrochemical prelithiation compensates for first-cycle lithium loss, improving energy efficiency and capacity retention.

### 5.5 Optimized operating conditions

Careful control of voltage ranges (2.8–4.2 V), moderate charge/discharge rates (≤0.5C), operating temperatures (25–40 °C), and stack pressure (∼5–10 MPa) reduces uneven lithium deposition and SEI degradation.

### 5.6 Advanced characterization and modeling

*In situ*/*operando* techniques (cryo-TEM, X-ray tomography, Raman spectroscopy) combined with computational modeling (DFT, molecular dynamics, phase-field) provide real-time insights into lithium nucleation, SEI evolution, and dendrite formation, guiding rational design of materials and interfaces.

### 5.7 Integrated strategy for AFLMBs

Combining ultrathin protective interlayers, DN-modulated LHCEs, artificial SEI layers, prelithiation, and solid-state electrolytes delivers synergistic improvements in CE, cycle life, safety, and energy density. Standardized benchmarking under realistic conditions (high loading, fast charging, temperature variation) is critical for practical application.

## 6 Forward-looking research directions

Despite recent progress, major bottlenecks remain in interfacial stability at high cathode loadings, first-cycle lithium loss, and long-term cycling of solid-state electrolytes. Future research should focus on synergistic approaches that integrate ultrathin interface layers, DN-modulated LHCEs, and scalable prelithiation, while engineering SSE interfaces for all-solid-state AFLMBs to achieve mechanical robustness and high ionic conductivity. Integrating predictive modeling with *operando* characterization will enable rational material and interface design, while standardized metrics for Coulombic efficiency, overpotential, and capacity retention under realistic conditions will ensure reproducibility and accelerate the transition from laboratory studies to practical, high-performance anode-free batteries.

## Author contributions

Zewdu Tadesse: conceptualization, writing – original draft and editing. Haojie Fei: review and editing. Nikhitha Joseph: review and editing, and Petr Sáha: review & editing, supervision.

## Conflicts of interest

The authors declare that they have no known competing financial interests or personal relationships that could have appeared to influence the work reported in this paper.

## Data Availability

No primary research results, software or code have been included and no new data were generated or analysed as part of this review.

## References

[cit1] Chen W., Salvatierra R. V., Ren M., Chen J., Stanford M. G., Tour J. M. (2020). Adv. Mater..

[cit2] Afzali P., Gibertini E., Magagnin L. (2024). Electrochim. Acta.

[cit3] Chen Y., Mao Y., Hao X., Cao Y., Wang W. (2021). ChemElectroChem.

[cit4] Heubner C., Maletti S., Auer H., Hüttl J., Voigt K., Lohrberg O., Nikolowski K., Partsch M., Michaelis A. (2021). Adv. Funct. Mater..

[cit5] Liu S., Yu X., Yan Y., Zeng T., Wang X., Tian G., Wang C., Wang S., Zeng Y., Shu C. (2023). Energy Storage Mater..

[cit6] Liu X., Liu J., Zhao H., Dong C., Liu F., Li L. (2024). J. Colloid Interface Sci..

[cit7] Ma C., Li R., Li C., Zhou J., He Y., Jiao F. (2024). Energy Storage Mater..

[cit8] Sun Z., Wang Y., Qin Y., Yang P., Wu H., Li X., Hu X., Xiao C., Zhao H., Ma M., Su Y., Ding S. (2023). Energy Storage Mater..

[cit9] Wang T., Mao Y., Wang J., Sun C. (2023). ACS Appl. Mater. Interfaces.

[cit10] Zhang Y., Zuo T.-T., Popovic J., Lim K., Yin Y.-X., Maier J., Guo Y.-G. (2020). Mater. Today.

[cit11] Shi P., Zhang X.-Q., Shen X., Zhang R., Liu H., Zhang Q. (2020). Adv. Mater. Technol..

[cit12] Yang C., Fu K., Zhang Y., Hitz E., Hu L. (2017). Adv. Mater..

[cit13] Wondimkun Z. T., Tegegne W. A., Shi-Kai J., Huang C.-J., Sahalie N. A., Weret M. A., Hsu J.-Y., Hsieh P.-L., Huang Y.-S., Wu S.-H., Su W.-N., Hwang B. J. (2021). Energy Storage Mater..

[cit14] Wondimkun Z. T., Beyene T. T., Weret M. A., Sahalie N. A., Huang C.-J., Thirumalraj B., Jote B. A., Wang D., Su W.-N., Wang C.-H., Brunklaus G., Winter M., Hwang B.-J. (2020). J. Power Sources.

[cit15] Zhu Y., Cui Y., Alshareef H. N. (2021). Nano Lett..

[cit16] Peng M.-J., Zhou J.-Q., Han T.-T., Zhou Y., Liu J., Xu N., Wang Z.-K., Lin W.-B., Yan C.-L. (2024). Rare Met..

[cit17] Wang Y., Noguchi H. (2023). Batter. Supercaps.

[cit18] Wang Y., Qu Z., Geng S., Liao M., Ye L., Shadike Z., Zhao X., Wang S., Xu Q., Yuan B., Zhang X., Gao X., Jiang X., Peng H., Sun H. (2023). Angew Chem. Int. Ed. Engl..

[cit19] Cho S., Kim D. Y., Lee J. I., Kang J., Lee H., Kim G., Seo D. H., Park S. (2022). Adv. Funct. Mater..

[cit20] Chen L., Chiang C.-L., Zeng G., Tang Y., Wu X., Zhou S., Zhang B., Zhang H., Yan Y., Liu T., Liao H.-G., Wang C., Kuai X., Lin Y.-G., Qiao Y., Sun S.-G. (2023). J. Mater. Chem. A.

[cit21] Dong L., Zhang S., Song D., Liu Y., Yang C. (2023). J. Chem. Eng..

[cit22] Fuchs T., Becker J., Haslam C. G., Lerch C., Sakamoto J., Richter F. H., Janek J. (2022). Adv. Energy Mater..

[cit23] Fuchs T., Ortmann T., Becker J., Haslam C. G., Ziegler M., Singh V. K., Rohnke M., Mogwitz B., Peppler K., Nazar L. F., Sakamoto J., Janek J. (2024). Nat. Mater..

[cit24] Hasegawa T., Bai F., Mori D., Taminato S., Takeda Y., Yamamoto O., Izumi H., Minami H., Imanishi N. (2022). ChemElectroChem.

[cit25] Hawari N. H., Huang X., Butarbutar L. M., Prayogi A., Hidayat H. N., Sumboja A., Ding N. (2024). J. Alloys Compd..

[cit26] Hu F., Chen J., Cao H., Wang H., Guo H., Ouyang X. (2024). Adv. Funct. Mater..

[cit27] Huang C. J., Hsu Y. C., Shitaw K. N., Siao Y. J., Wu S. H., Wang C. H., Su W. N., Hwang B. J. (2022). ACS Appl. Mater. Interfaces.

[cit28] Huang C. J., Thirumalraj B., Tao H. C., Shitaw K. N., Sutiono H., Hagos T. T., Beyene T. T., Kuo L. M., Wang C. C., Wu S. H., Su W. N., Hwang B. J. (2021). Nat. Commun..

[cit29] Zhu Y., Wu S., Zhang L., Zhang B., Liao B. (2023). ACS Appl. Mater. Interfaces.

[cit30] Yu D., Lee C., Wang W., Miyahara Y., Miyazaki K., Abe T. (2023). Electrochim. Acta.

[cit31] Wu W., Ning D., Zhang J., Liu G., Zeng L., Yao H., Wang M., Deng L., Yao L. (2023). Energy Storage Mater..

[cit32] Vanaphuti P., Su L., Manthiram A. (2024). Small Methods.

[cit33] Huang S., Lu S., Lv Y., Li N., Wu Z., Zhong G., Ren X., Wang Y., Sun B., Huang Y., Kang F., Cao Y. (2023). Nano Res..

[cit34] Huang Y. K., Chen H., Nyholm L. (2023). Small.

[cit35] Kim J. G., Gu D., Cho K. H., Im C. Y., Kim S. J. (2023). Small.

[cit36] Kim S., Lee M., Oh S., Ryu W.-H. (2023). J. Chem. Eng..

[cit37] Lee J., Kim J., Lee D. G., Son D., Lee J., Kim S., Han S., Choi N.-S., Lee T. K., Lee J. (2024). J. Chem. Eng..

[cit38] Lin L., Qin K., Hu Y. S., Li H., Huang X., Suo L., Chen L. (2022). Adv. Mater..

[cit39] Ouyang Z., Wang Y., Wang S., Geng S., Zhao X., Zhang X., Xu Q., Yuan B., Tang S., Li J., Wang F., Yao G., Sun H. (2024). Adv. Mater..

[cit40] Ma C., Weng S., Zhang Y., Zhang X., Liu T., Liu L., Zhao Z., Liu C., Zhao Z., Wang X., Wu B., Mu D., Wu F. (2022). Nano Lett..

[cit41] Pande V., Viswanathan V. (2019). ACS Energy Lett..

[cit42] Jo C.-H., Sohn K.-S., Myung S.-T. (2023). Energy Storage Mater..

[cit43] Lin G., Meng T., Peng Y., Li P., Hu X. (2024). Small Methods.

[cit44] Marrache R., Peled E. (2024). J. Electrochem. Soc..

[cit45] Wang H., Xie Z., Liu C., Hu B., Liao S., Yan X., Ye F., Huang S., Guo Y., Ouyang C. (2023). ACS Appl. Mater. Interfaces.

[cit46] Du J., Gao C., Feng T. (2022). Future Internet.

[cit47] Tang K., Tian L., Zhang Y., Xu Z. J. (2024). J. Mater. Chem. A.

[cit48] Wang D., Qiu J., Inui N., Hagiwara R., Hwang J., Matsumoto K. (2023). ACS Energy Lett..

[cit49] Qiu F., Li X., Deng H., Wang D., Mu X., He P., Zhou H. (2019). Adv. Energy Mater..

[cit50] Salvatierra R. V., Chen W., Tour J. M. (2021). Adv. Energy Sustain. Res..

[cit51] Li Y., Zhang Y., Li Z., Yan Z., Xiao X., Liu X., Chen J., Shen Y., Sun Q., Huang Y. (2022). Adv. Sci..

[cit52] Ping Zheng Y., Hu X. J., Xu X. M., Wang H. F., Sun Y. L., Liu T., Bin Liu X., Wu Z. P., Xia B. Y. (2024). Batter. Supercaps.

[cit53] Zeng X., Mahato M., Oh W., Yoo H., Nguyen V. H., Oh S., Valurouthu G., Jeong S. K., Ahn C. W., Gogotsi Y., Oh I. K. (2024). Energy Environ. Mater..

[cit54] Weldeyohannes H. H., Abrha L. H., Nikodimos Y., Shitaw K. N., Hagos T. M., Huang C.-J., Wang C.-H., Wu S.-H., Su W.-N., Hwang B. J. (2021). J. Power Sources.

[cit55] Wang S., Wang Y., Ouyang Z., Geng S., Chen Q., Zhao X., Yuan B., Zhang X., Tang S., Xu Q., Chen P., Peng H., Sun H. (2025). Nat. Mater..

[cit56] Lee J., Kim J., Jang W., Lee D. G., Kim H., An Y., Son J., Kang M., Lee G., Lee J., Son D., Park C.-Y., Choi K., Shin D., Lee T. K., Moon J., Im S. G., Lee J. (2026). Joule.

[cit57] Zhao Y., Li S., Zhu L., Li Y., Xu S., Dou H., Zhang X. (2025). Chem. Sci..

[cit58] Yu H., Sun P., Cheng H., Ding Z., Luo D. (2025). Electrochim. Acta.

[cit59] Huang C.-J., Thirumalraj B., Tao H.-C., Shitaw K. N., Sutiono H., Hagos T. T., Beyene T. T., Kuo L.-M., Wang C.-C., Wu S.-H., Su W.-N., Hwang B. J. (2021). Nat. Commun..

[cit60] Lee J.-A., Kang H., Kim S., Lee K., Byun J. H., Kwon E., Seo S., Kwak K., Ryu K. H., Choi N.-S. (2024). Energy Storage Mater..

[cit61] Merso S. K., Tekaligne T. M., Weldeyohannes H. H., Nikodimos Y., Shitaw K. N., Jiang S.-K., Huang C.-J., Wondimkun Z. T., Jote B. A., Wichmann L., Brunklaus G., Winter M., Wu S.-H., Su W.-N., Mou C.-Y., Hwang B. J. (2022). J. Energy Storage.

[cit62] Khramenkova A. V., Moshchenko V. V., Laptii P. V., Finaeva O. A., Evstigneeva M. A., Chernyavsky V. A., Maximov M. Y. (2024). Appl. Phys. A.

[cit63] Koul S., Morita Y., Fujisaki F., Ogasa H., Fujiwara Y., Kushima A. (2022). J. Electrochem. Soc..

[cit64] Zhong G., Ma J., Li N., Yin R., Jia T., Cai K., Kang F., Cao Y. (2024). Carbon.

[cit65] Zhou J., Cheng Y., Chen B., Shi C., He F., Zhao N., He C. (2026). Angew. Chem., Int. Ed..

[cit66] Mao M., Ji X., Wang Q., Lin Z., Li M., Liu T., Wang C., Hu Y. S., Li H., Huang X., Chen L., Suo L. (2023). Nat. Commun..

[cit67] Deng J., Lin H., Hu L., Zhan C., Weng Q., Yu X., Sun X., Zhang Q., Mo J., Li B. (2024). Molecules.

[cit68] Hagos T. M., Berhe G. B., Hagos T. T., Bezabh H. K., Abrha L. H., Beyene T. T., Huang C.-J., Yang Y.-W., Su W.-N., Dai H., Hwang B.-J. (2019). Electrochim. Acta.

[cit69] Zhang J., Zhang H., Deng L., Yang Y., Tan L., Niu X., Chen Y., Zeng L., Fan X., Zhu Y. (2023). Energy Storage Mater..

[cit70] Jote B. A., Beyene T. T., Sahalie N. A., Weret M. A., Olbassa B. W., Wondimkun Z. T., Berhe G. B., Huang C.-J., Su W.-N., Hwang B. J. (2020). J. Power Sources.

[cit71] Zhang X., Huang L., Xie B., Zhang S., Jiang Z., Xu G., Li J., Cui G. (2023). Adv. Energy Mater..

[cit72] Beyene T. T., Jote B. A., Wondimkun Z. T., Olbassa B. W., Huang C. J., Thirumalraj B., Wang C. H., Su W. N., Dai H., Hwang B. J. (2019). ACS Appl. Mater. Interfaces.

[cit73] Chang W., Xu T., Steingart D. (2022). J. Electrochem. Soc..

[cit74] Genovese M., Louli A. J., Weber R., Martin C., Taskovic T., Dahn J. R. (2019). J. Electrochem. Soc..

[cit75] Zhang Y., Liu J., Li Y., Zhao D., Huang W., Zheng Y., Zhou J., Zhu C., Deng C., Sun Y., Qian T., Yan C. (2023). Adv. Funct. Mater..

[cit76] Zhou C., Samson A. J., Garakani M. A., Thangadurai V. (2021). J. Electrochem. Soc..

[cit77] Qian J., Adams B. D., Zheng J., Xu W., Henderson W. A., Wang J., Bowden M. E., Xu S., Hu J., Zhang J. G. (2016). Adv. Funct. Mater..

[cit78] Zegeye T. A., Su W.-N., Fenta F. W., Zeleke T. S., Jiang S.-K., Hwang B. J. (2020). ACS Appl. Energy Mater..

[cit79] Shin W., Manthiram A. (2022). ACS Appl. Mater. Interfaces.

[cit80] Kim S., Didwal P. N., Fiates J., Dawson J. A., Weatherup R. S., De Volder M. (2024). ACS Energy Lett..

[cit81] Lin X., Shen Y., Yu Y., Huang Y. (2024). Adv. Energy Mater..

[cit82] Wei T., Lu J., Zhang P., Yang G., Sun C., Zhou Y., Zhuang Q., Tang Y. (2023). Chin. Chem. Lett..

[cit83] Yoon J. S., Liao D. W., Greene S. M., Cho T. H., Dasgupta N. P., Siegel D. J. (2024). ACS Appl. Mater. Interfaces.

[cit84] You X., Feng Y., Ning D., Yao H., Wang M., Wang J., Chen B., Zhong G. H., Yang C., Wu W. (2024). Nano Lett..

[cit85] Zhou J., Qin J., Zhan H. (2024). Chemphyschem.

[cit86] Ouyang Z., Wang S., Wang Y., Muqaddas S., Geng S., Yao Z., Zhang X., Yuan B., Zhao X., Xu Q., Tang S., Zhang Q., Li J., Sun H. (2024). Adv. Mater..

[cit87] Zhang S., Liao H., Liu Z.-Q., Yan C., Huang J.-Q. (2024). Chin. Chem. Lett..

[cit88] Zhang S., Zeng J., Ma Y., Zhao Y., Qian Y., Suo L., Huang J., Wang X., Li W., Zhang B. (2023). Electrochim. Acta.

[cit89] Li J., Ma Z., Yang K., Zhao F., Yang H., Wang H., He Y. (2025). J. Colloid Interface Sci..

[cit90] Li N., Jia T., Liu Y., Ouyang Y., Lv Y., Zhong G., Wang Y., Sun B., Lu S., Huang S., Kang F., Cao Y. (2023). Mater. Today Energy.

[cit91] Lo C.-A., Chang C.-C., Tsai Y.-W., Jiang S.-K., Hwang B. J., Mou C.-Y., Wu H.-L. (2021). ACS Appl. Energy Mater..

[cit92] Lu G., Dong Z., Liu W., Jiang X., Yang Z., Liu Q., Yang X., Wu D., Li Z., Zhao Q., Hu X., Xu C., Pan F. (2021). Sci. Bull..

[cit93] Sahalie N. A., Wondimkun Z. T., Su W.-N., Weret M. A., Fenta F. W., Berhe G. B., Huang C.-J., Hsu Y.-C., Hwang B. J. (2020). ACS Appl. Energy Mater..

[cit94] Wang J., Yang P., Wang Y., Wang S. (2024). Carbon.

[cit95] Zhao X., Yang Q., Quan Z. (2019). ChemCommun.

[cit96] Zhang S. S., Fan X., Wang C. (2017). Electrochim. Acta.

[cit97] Li Y., Bu M., Mu C., Yang C. (2024). Mater. Lett..

[cit98] Cho K.-Y., Hong S.-H., Kwon J., Song H., Kim S., Jo S., Eom K. (2021). Appl. Surf. Sci..

[cit99] Mirbagheri S., Gibertini E., Magagnin L. (2024). Batteries.

[cit100] Pyo S., Ryu S., Gong Y. J., Cho J., Yun H., Kim H., Lee J., Min B., Choi Y., Yoo J., Kim Y. S. (2022). Adv. Energy Mater..

[cit101] Li X., Su Y., Qin Y., Huang F., Mei S., He Y., Peng C., Ding L., Zhang Y., Peng Y., Deng Z. (2023). Adv. Mater..

[cit102] Zhang D., Dai A., Fan B., Li Y., Shen K., Xiao T., Hou G., Cao H., Tao X., Tang Y. (2020). ACS Appl. Mater. Interfaces.

[cit103] Nguyen M. H., Kim D., Kim B. K., Park S. (2024). Adv. Funct. Mater..

[cit104] Guan W., Lv Y., Ma C., Zhang Q., Wei A., Liu X. (2023). Mater. Chem. Front..

[cit105] Koul S., Morita Y., Ogasa H., Fujiwara Y., Kushima A. (2020). ECS Meet. Abstr..

[cit106] Lin L., Suo L., Hu Y. s., Li H., Huang X., Chen L. (2021). Adv. Energy Mater..

[cit107] Sun J., Zhang S., Li J., Xie B., Ma J., Dong S., Cui G. (2023). Adv. Mater..

[cit108] Yu K., Chen J., xie X., Lin K., Li J., Shi Z. (2022). Surf. Interfaces.

[cit109] Abrha L. H., Zegeye T. A., Hagos T. T., Sutiono H., Hagos T. M., Berhe G. B., Huang C.-J., Jiang S.-K., Su W.-N., Yang Y.-W., Hwang B.-J. (2019). Electrochim. Acta.

[cit110] Temesgen N. T., Tegegne W. A., Shitaw K. N., Fenta F. W., Nikodimos Y., Taklu B. W., Jiang S.-K., Huang C.-J., Wu S.-H., Su W.-N., Hwang B. J. (2021). J. Taiwan Inst. Chem. Eng..

[cit111] Merso S. K., Tekaligne T. M., Adigo Weret M., Shitaw K. N., Nikodimos Y., Yang S.-C., Muche Z. B., Taklu B. W., Hotasi B. T., Chang C.-Y., Jiang S.-K., Brunklaus G., Winter M., Wu S.-H., Su W.-N., Mou C.-Y., Hwang B. J. (2024). J. Chem. Eng..

[cit112] Pathirana T., Rakov D. A., Chen F., Forsyth M., Kerr R., Howlett P. C. (2021). ACS Appl. Energy Mater..

[cit113] Che H., Chen S., Xie Y., Wang H., Amine K., Liao X.-Z., Ma Z.-F. (2017). Energy Environ. Sci..

[cit114] Wu X., Pan K., Jia M., Ren Y., He H., Zhang L., Zhang S. (2019). Green Energy Environ..

[cit115] Zhou B., Stoševski I., Bonakdarpour A., Wilkinson D. P. (2023). Adv. Funct. Mater..

[cit116] van der Lubbe S. C. C., Canepa P. (2023). npj Comput. Mater..

[cit117] Lv L., Wang Y., Huang W., Wang Y., Zhu G., Zheng H. (2022). Electrochim. Acta.

[cit118] Shen X., Zhang R., Chen X., Cheng X.-B., Li X., Zhang Q. (2020). Adv. Energy Mater..

[cit119] Sayavong P., Zhang W., Oyakhire S. T., Boyle D. T., Chen Y., Kim S. C., Vilá R. A., Holmes S. E., Kim M. S., Bent S. F., Bao Z., Cui Y. (2023). J. Am. Chem. Soc..

[cit120] Ren J., Zhang S., Niu M., Dong Y., Liang L., Zhang S., Zhao L. L., Dong L., Yang C., Liang J.-Y. (2024). ACS Appl. Energy Mater..

[cit121] Ho V.-C., Ngo D. T., Le H. T. T., Verma R., Kim H.-S., Park C.-N., Park C.-J. (2018). Electrochim. Acta.

[cit122] Sahalie N. A., Assegie A. A., Su W.-N., Wondimkun Z. T., Jote B. A., Thirumalraj B., Huang C.-J., Yang Y.-W., Hwang B.-J. (2019). J. Power Sources.

[cit123] Qin K., Nguyen J. V., Yang Z., Luo C. (2023). Mater. Today Energy.

[cit124] Bertoli L., Bloch S., Andersson E., Magagnin L., Brandell D., Mindemark J. (2023). Electrochim. Acta.

[cit125] Hawari N. H., Xie H., Prayogi A., Sumboja A., Ding N. (2023). RSC Adv..

[cit126] Kang D. W., Moon J., Choi H.-Y., Shin H.-C., Kim B. G. (2021). J. Power Sources.

[cit127] Hagos T. T., Thirumalraj B., Huang C. J., Abrha L. H., Hagos T. M., Berhe G. B., Bezabh H. K., Cherng J., Chiu S. F., Su W. N., Hwang B. J. (2019). ACS Appl. Mater. Interfaces.

[cit128] Li H., Yan C., Wang S. (2025). EcoEnergy.

[cit129] Hagos T. T., Su W.-N., Huang C.-J., Thirumalraj B., Chiu S.-F., Abrha L. H., Hagos T. M., Bezabh H. K., Berhe G. B., Tegegne W. A., Cherng J.-Y., Yang Y.-W., Hwang B.-J. (2020). J. Power Sources.

[cit130] Hagos T. M., Hagos T. T., Bezabh H. K., Berhe G. B., Abrha L. H., Chiu S.-F., Huang C.-J., Su W.-N., Dai H., Hwang B. J. (2020). ACS Appl. Energy Mater..

[cit131] Hotasi B. T., Hagos T. M., Huang C. J., Jiang S.-K., Jote B. A., Shitaw K. N., Bezabh H. K., Wang C.-H., Su W.-N., Wu S.-H., Hwang B. J. (2022). J. Power Sources.

[cit132] Hagos T. M., Bezabh H. K., Redda H. G., Moges E. A., Huang W.-H., Huang C.-J., Su W.-N., Dai H., Hwang B. J. (2021). J. Power Sources.

[cit133] Redda H. G., Nikodimos Y., Su W.-N., Chen R.-S., Hagos T. M., Bezabh H. K., Weldeyohannes H. H., Hwang B. J. (2022). Mater. Today Energy.

[cit134] Zhou C., Zheng L., He T., Garakani M. A., Abouali S., Shen Y., Chen L., Thangadurai V. (2021). Energy Storage Mater..

[cit135] Agnihotri T., Ahmed S. A., Tamilarasan E. B., Hasan R., Hotasi B. T., Bezabh H. K., Suwito S., Nikodimos Y., Jiang S.-K., Shitaw K. N., Muche Z. B., Huang P. Y., Lee Y.-C., Su W.-N., Wu S.-H., Hwang B. J. (2024). J. Chem. Eng..

[cit136] Temesgen N. T., Bezabh H. K., Weret M. A., Shitaw K. N., Nikodimos Y., Taklu B. W., Lakshmanan K., Yang S.-C., Jiang S.-K., Huang C.-J., Wu S.-H., Su W.-N., Hwang B. J. (2023). J. Power Sources.

[cit137] He L., Sun Q., Lu L., Adams S. (2021). ACS Appl. Mater. Interfaces.

[cit138] Louli A. J., Coon M., Genovese M., deGooyer J., Eldesoky A., Dahn J. R. (2021). J. Electrochem. Soc..

[cit139] Lim H.-S., Nguyen D. T., Lochala J. A., Cao X., Zhang J.-G. (2024). ACS Energy Lett..

[cit140] Cai K., Zhang M., Zhong G., Kang G., Biao J., Li C., Liu Y., Zhou G., Kang F., Cao Y. (2024). J. Mater. Chem. A.

[cit141] Chang W., Thorsteinsson G., Janakiraman U., Chowdhury R. R., Herman Z., Katzman L., Steingart D. A. (2024). J. Electrochem. Soc..

[cit142] Zhang S. S. (2018). ACS Appl. Energy Mater..

[cit143] Liu W., Luo Y., Hu Y., Chen Z., Wang Q., Chen Y., Iqbal N., Mitlin D. (2023). Adv. Energy Mater..

[cit144] Gao X., Zhou Y.-N., Han D., Zhou J., Zhou D., Tang W., Goodenough J. B. (2020). Joule.

[cit145] Maraschky A., Akolkar R. (2020). J. Electrochem. Soc..

[cit146] Wu B., Chen C., Raijmakers L. H. J., Liu J., Danilov D. L., Eichel R.-A., Notten P. H. L. (2023). Energy Storage Mater..

[cit147] Hatzell K. B. (2023). ACS Energy Lett..

[cit148] Vishweswariah K., Ningappa N. G., Bouguern M. D., Kumar M R A., Armand M. B., Zaghib K. (2025). Adv. Energy Mater..

[cit149] Ju Y., Kim B., Youn B., Song Y., Jang H., Lee D. (2025). J. Energy Storage.

[cit150] Li C., Lu G., Wu X., Zhang M., Wu X., Piao Z., Xiao X., Han Z., Zhong X., Qiao Q., Chen B., Wang Y., Zhou G., Cheng H.-M. (2026). Nat. Synth..

[cit151] Chen B., Zhang H., Xuan J., Offer G. J., Wang H. (2020). Adv. Mater. Technol..

[cit152] Harrison K. L., Merrill L. C., Long D. M., Randolph S. J., Goriparti S., Christian J., Warren B., Roberts S. A., Harris S. J., Perry D. L., Jungjohann K. L. (2021). iScience.

[cit153] Liu Z., Bai S., Burke S., Burrow J. N., Geurts R., Huang C.-J., Jiao C., Lee H.-B., Meng Y. S., Novák L., Winiarski B., Wang J., Wu K., Zhang M. (2025). Chem. Rev..

[cit154] Peng Y., Nishikawa K. (2025). Cell Rep. Phys. Sci..

[cit155] Meddings N., Heinrich M., Overney F., Lee J.-S., Ruiz V., Napolitano E., Seitz S., Hinds G., Raccichini R., Gaberšček M., Park J. (2020). J. Power Sources.

[cit156] Krishna D. N. G., Philip J. (2022). Appl. Surf. Sci. Adv..

[cit157] Wu Z.-Y., Deng L., Li J.-T., Zanna S., Seyeux A., Huang L., Sun S.-G., Marcus P., Światowska J. (2022). Journal.

[cit158] Gober M., Amai J., Torres J. R., Zhang Y., Bilheux J.-C., Bilheux H. Z., Demeneghi G., Tang S., Smith G., Nelson G. J. (2025). J. Power Sources.

[cit159] Sau S., Srivastava S., Panda M., Sagdeo A., Mitra S. (2024). J. Power Sources.

[cit160] Xiang S., Zhu L., Fu L., Wang M., Zhang X., Tang Y., Sun D., Wang H. (2025). eScience.

[cit161] Lindsey I., Mondl C., Meng X. (2026). Energy Adv..

